# Physical Layer Security in Two-Way SWIPT Relay Networks with Imperfect CSI and a Friendly Jammer

**DOI:** 10.3390/e25010122

**Published:** 2023-01-06

**Authors:** Maymoona Hayajneh, Thomas Aaron Gulliver

**Affiliations:** Department of Electrical and Computer Engineering, University of Victoria, P.O. Box 1700, STN CSC, Victoria, BC V8W 2Y2, Canada

**Keywords:** amplify and forward, eavesdropper, imperfect channel state information, relay, secrecy capacity, simultaneous wireless information and power transfer, time switching, two-way

## Abstract

In this paper, the security of two-way relay communications in the presence of a passive eavesdropper is investigated. Two users communicate via a relay that depends solely on energy harvesting to amplify and forward the received signals. Time switching is employed at the relay to harvest energy and obtain user information. A friendly jammer is utilized to hinder the eavesdropping from wiretapping the information signal. The eavesdropper employs maximal ratio combining and selection combining to improve the signal-to-noise ratio of the wiretapped signals. Geometric programming (GP) is used to maximize the secrecy capacity of the system by jointly optimizing the time switching ratio of the relay and transmit power of the two users and jammer. The impact of imperfect channel state information at the eavesdropper for the links between the eavesdropper and the other nodes is determined. Further, the secrecy capacity when the jamming signal is not perfectly cancelled at the relay is examined. The secrecy capacity is shown to be greater with a jammer compared to the case without a jammer. The effect of the relay, jammer, and eavesdropper locations on the secrecy capacity is also studied. It is shown that the secrecy capacity is greatest when the relay is at the midpoint between the users. The closer the jammer is to the eavesdropper, the higher the secrecy capacity as the shorter distance decreases the signal-to-noise ratio of the jammer.

## 1. Introduction

There has been a shift in wireless network research from spectral efficiency and quality of service (QoS) constraints to energy efficiency and green communication [1] to reduce the power consumption [2]. Green energy resources such as solar, wind, thermal, and mechanical vibrations can be employed to improve the energy efficiency of energy-constrained devices such as in wireless sensor networks. Energy harvesting (EH) to convert the available energy in the surrounding area into electricity has been the subject of recent research [3]. Energy harvesting from radio frequency (RF) signals has been employed in wireless communication systems to prolong the lifetime of devices in energy-constrained systems [4]. Wireless power transmission (WPT) for EH is a promising solution to sustainable energy for wireless devices [5,6,7]. It can provide a reliable source of energy for devices that are difficult to service due to mobility and location [8,9,10].

RF signals can carry both information and energy, so WPT in wireless communication systems is known as simultaneous wireless information and power transfer (SWIPT) [6,8,11,12,13]. Two circuits are usually employed to harvest energy and retrieve information [14]. Two SWIPT protocols have been developed, power splitting (PS) and time switching (TS) [15]. With TS, the receiver switches between the two circuits, while in PS, a fraction of the signal is directed to the EH circuit, and the remaining part is sent to the information retrieval circuit. The maximum transmission rate using optimal PS and TS was derived in [16,17], respectively. In [16], the outage probability was obtained for a decode and forward (DF) relay network, and the optimal transmission rates with PS and TS were determined. A SWIPT-enabled relay was considered in [17] for three scenarios, ideal (simultaneous EH and information retrieval), PS, and TS, and the maximum rates for each were obtained. PS and TS can be used separately or combined as a hybrid protocol, where the relay switches between PS and TS [18]. The optimal TS and PS ratios were derived to maximize the throughput with an EH relay, and the hybrid protocol was shown to provide the best performance. In [19,20], joint PS and TS schemes were considered for amplify and forward (AF) and DF relay networks, respectively. In [19], the outage probability, energy efficiency, and network throughput were derived as a function of the PS and TS ratios, and the network throughput was maximized. In [20], two optimization problems were jointly formulated to minimize the outage probability. These outage probabilities were shown to be better than that with the hybrid protocol in [18]. The system throughput of a cognitive two-way relay network was maximized in [21] using an optimal offline joint relay selection and power allocation scheme.

Wireless signals are more vulnerable to eavesdropping compared to wired signals given the broadcast nature of wireless systems. The physical layer security of the wiretap channel was introduced in [22] and is defined as the difference between the capacity of the link between the source and destination and the capacity of the wiretap link between the source and eavesdropper. This can be used to assist upper-layer cryptographic techniques [23,24,25]. Physical-layer-security-based solutions exploit the physical properties of wireless channels, such as fading and interference, to secure transmissions between users in the presence of eavesdroppers [26,27].

Physical layer security has been considered for relay networks [28], cellular networks [29,30], cognitive radio networks [31], Internet of Things (IoT) networks [32], and massive multiple-input multiple-output (MIMO) networks [33]. However, wireless channel conditions have a significant effect on the solutions [34]. Physical layer security with cooperative relaying has been employed to overcome this issue [35]. This was first studied in [36] for an untrusted relay network, which was considered as a possible eavesdropper. One-way communications was examined in [37] for DF and AF EH relays, and it was shown that DF outperforms AF in terms of secrecy performance. The secrecy capacity was analysed in [38] for PS and TS relaying protocols in a one-way untrusted relay network, and PS outperformed TS.

Two-way relay channels in which two users simultaneously exchange messages were first considered in [39] and more recently in [40]. The spectral efficiency with two-way relaying is higher than with one-way relaying. In [41], a two-way EH-based relay network with an eavesdropper was investigated. The secrecy capacity was maximized and an iterative method employed to obtain the optimal TS and PS ratios for high signal-to-noise ratios (SNRs) based on the instantaneous channel state information (CSI). It was shown that near-optimal secrecy capacity is achievable with the proposed approach even when the wiretapped channels are unknown. Joint secrecy capacity and energy efficiency were considered in [42] for a two-way untrusted relay network. The probability of successful eavesdropping in a two-way EH DF relay network was derived in [43] assuming independent κ-μ shadowed fading. It was shown that allocating extra power for information decoding over a small reception time improves the secrecy capacity. In [44], the intercept probability was derived for a two-way DF EH relay network in the presence of multiple eavesdroppers. The effect of the PS factor on the secrecy capacity was studied. The secrecy capacity of a two-way communication network with multi-antenna time-switching relays in the presence of an eavesdropper was maximized in [45]. It this case, the secrecy capacity with equal transmit power is better than with unequal transmit power.

Cooperative jamming can improve the secrecy capacity [46,47,48]. Friendly jamming (FJ) and Gaussian noise jamming (GNJ) have been considered to improve the secrecy capacity of wireless communication networks. The jamming signal is known at the receiver when FJ is used [24], while with GNJ, the jamming signal is considered to be noise at the receiver [49]. While both FJ and GNJ can improve the secrecy capacity, the performance with FJ is better because the users can cancel this signal. In [50], a system with two eavesdroppers and an EH friendly jammer was considered. One eavesdropper is near the user, while the other is near the jammer, and they cooperate to obtain user signals and mitigate the effects of jamming. The secrecy capacity and energy efficiency of the network were maximized by optimizing the jamming signal power. In [51], the secrecy capacity with a friendly jammer was investigated for a one-way untrusted relay network with non-line-of-sight transmissions. A jammer was employed in [52] for an EH-based relay network to secure two-way communications, and a lower bound was derived for the secrecy capacity at high SNRs. It was shown that FJ with two-way communications outperforms one-way and two-way communications without jamming and with GNJ. In [53], the secrecy capacity of one-way untrusted relay communications was optimized considering the transmit and jamming powers with an EH relay threshold. The secrecy performance with untrusted EH relays and energy-aware distributed beamforming was investigated in [54]. The secrecy capacity was increased in [55] by choosing GNJ and relay nodes from multiple friendly, but selfish intermediate nodes. Price competition was used for power allocation to these nodes, and their profit to maximize the secrecy capacity was determined. In [56], a full-duplex jammer (FDJ) and half-duplex jammer (HDJ) were proposed to improve security while exploiting EH. An interference-limited scenario was considered, and closed-form cumulative distribution functions (CDFs) were derived for the signal-to-interference-plus-noise ratio at the destination and eavesdropper nodes.

A two-way untrusted relay system with multiple friendly jammers was considered in [57], and the jamming power was optimized to improve the secrecy capacity. In [58], a network with multiple relay–user pairs was investigated in the presence of multiple eavesdroppers. Joint relay–user pairs and friendly jammer selection were determined to maximize the secrecy capacity. The secrecy capacity was optimized in [57] using a Stackelberg game for power allocation between users and friendly jammers.

In [59], adaptive cooperative jamming in the presence of multiple eavesdroppers was investigated for an EH relay. The secrecy capacity was maximized by optimizing the power allocation factor. A two-way EH relay network with an eavesdroppers and a friendly jammer was considered in [60]. The optimal PS and TS factors were derived to maximize the secrecy capacity, and PS was shown to be better than TS. A two-way relay network with partial relay selection and hybrid PS and TS at the intermediate nodes was investigated in [61]. It was shown that secure communications are possible with an appropriate selection of the parameters.

In the results given above, perfect knowledge of the CSI for the user and relay signals at the eavesdropper was assumed. However, this is not a practical assumption considering unknown delays and channel estimation errors. In two-way relay networks, imperfect CSI results in imperfect self-interference cancellation [62]. In [63], a transmission scheme was proposed for multiple-input single-output (MISO) channels with imperfect CSI for the user and eavesdropper channels with cooperative jamming. In [64], the CSI for the channel between the jammer and eavesdropper was assumed to be unknown and imperfect CSI assumed between the jammer and user. The impact of imperfect CSI on the secrecy outage capacity with cooperative jamming was analysed. Although imperfect CSI has received some research attention, the impact of imperfect CSI on the security of a SWIPT two-way relay network has not yet been studied.

In this paper, the physical layer security of a two-way communication system with a relay employing TS to harvest energy, a friendly jammer, and imperfect CSI at a passive eavesdropper is studied. TS is simpler to implement than the PS considered in [65]. The eavesdropper employs maximal ratio combining (MRC) and selection combining (SC) to degrade the secrecy capacity. The power allocated to two users, a relay, and a jammer are jointly optimized in the presence of an eavesdropper with imperfect CSI. This system has not been previously considered in the literature for a TS EH relay. Furthermore, the effect of the imperfect cancellation of the jamming power at the relay is studied. The main contributions of this work are as follows:

1. The effect of channel estimation errors on the secrecy capacity is investigated when the eavesdropper employs MRC and SC. Imperfect CSI at the eavesdropper has not been previously considered.
2. The secrecy capacity is maximized by jointly optimizing the TS ratio and transmit powers of the two users and jammer.
3. The single condensation method (SCM) is used to convert the objective function into a standard geometric programming (GP) form. Then, GP is employed to transform the optimization problem into a convex form.
4. The effect of imperfect cancellation of the jamming signal at the relay is examined. This has not been considered previously in the literature.
5. The effect of the TS ratio on the secrecy capacity is investigated.
6. The secrecy capacity is evaluated with and without a jammer. In addition, results are given for different eavesdropper and jammer locations.

The remainder of this paper is organized as follows. The system model is given in Section 2. The secrecy capacity for the two-way relay network is presented in Section 3 for MRC and SC. In Section 4, the optimization problem is formulated and converted to a convex form. Section 5 presents the simulation results, and finally, some concluding remarks are given in Section 6.

## 2. System Model

**Figure 1:**
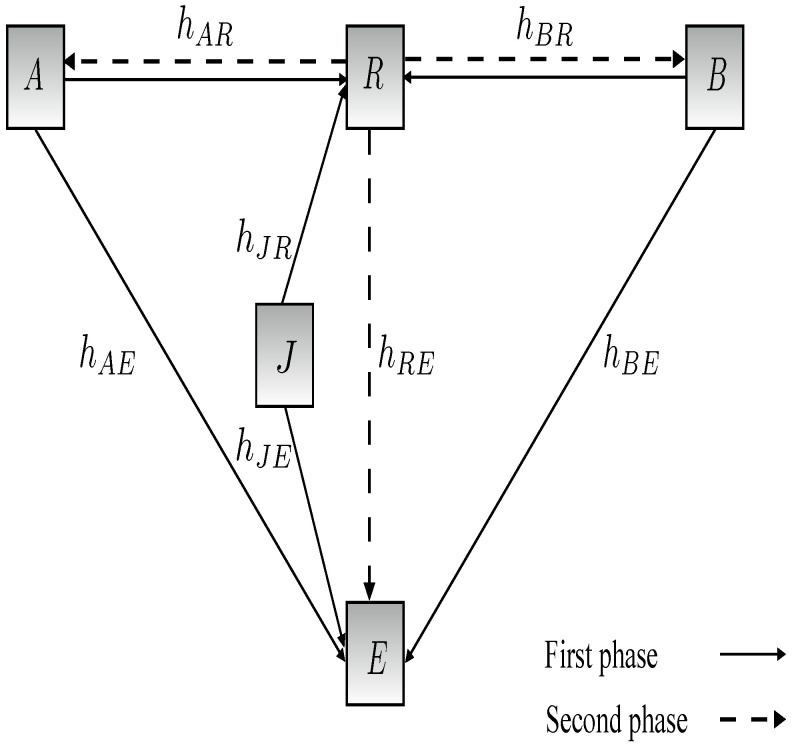
System model of a two-way wireless relay network with two users, a jammer, and an eavesdropper.

The two-way relay network considered here is shown in Figure 1. It consists of two users *A* and *B*, a trusted relay *R*, a friendly jammer *J*, and an eavesdropper *E*. Each of these nodes has a single antenna and operates in half-duplex mode. The friendly jammer can be another user node or a dedicated jamming node. The eavesdropper is randomly located near the relay to listen to the signals received by and transmitted from the relay. The *A*-*R*, *B*-*R*, *A*-*E*, *B*-*E*, *R*-*E*, *J*-*R*, and *J*-*E* channel links are denoted by $h_{AR}$, $h_{BR}$, $h_{AE}$, $h_{BE}$, $h_{RE}$, $h_{JR}$, and $h_{JE}$, respectively. Quasi-static fading channels are assumed so the channel gains, $h_{ij}$, are constant over the coherence time [15,41]. Rayleigh fading is assumed so the channel coefficients are Rayleigh random variables. Then, the channel gains $|h_{ij}{|}^{2}$ are exponentially distributed random variables with means λ and $\lambda_{Eve}$. The channels are assumed to be reciprocal such that $h_{ij}=h_{ji}$, $\left\{i,j\right\}\in \left\{A,B,R,J,E\right\},i\neq j$. The parameters $n_{A}$, $n_{B}$,$n_{R}$, and $n_{E}$ denote the additive white Gaussian noise (AWGN) at *A*, *B*, *R*, and *E*, respectively, with zero mean and variance $\sigma^{2}$.

In this paper, the practical case is considered where the channels at *A*, *B*, *R*, and *J* can be estimated accurately given that they are trusted nodes, but there are channel estimation errors at the eavesdropper [63]. The estimated channel gain from the eavesdropper to node *i*, $i\in \left\{A,B,R,J\right\},i\neq E$, is given by [62]

(1) $$h_{iE}={\hat{h}}_{iE}+e_{iE},$$

where ${\hat{h}}_{iE}$ is the estimated channel gain and $e_{iE}$ is the channel estimation error. For simplicity, denote $e_{iE}$ by $e_{E}$, which is a Gaussian distributed random variable with zero mean and variance $\sigma_{e}^{2}$. A summary of the notation used in this paper is given in Abbreviations.

**Figure 2:**
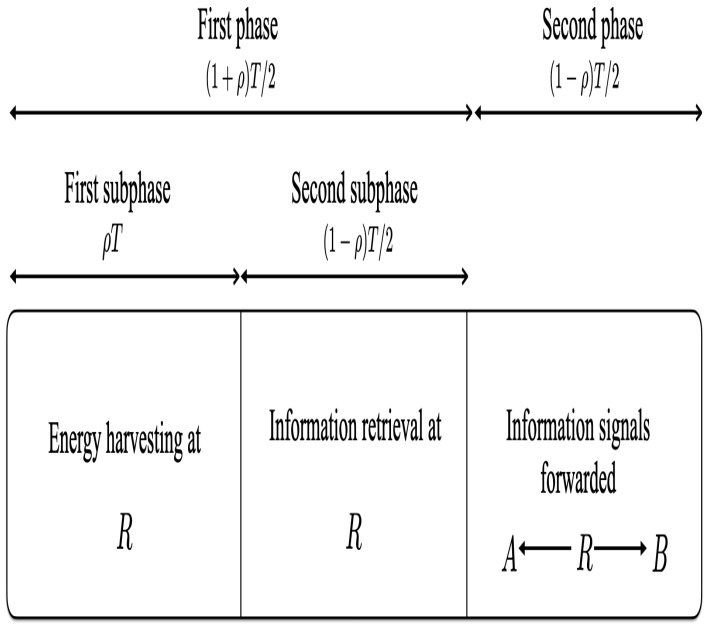
Transmission time frame for time switching (TS) in the two-way relay network.

Figure 2 illustrates the two phases required to forward signals between *A* and *B* in the relay network. The first phase is dedicated to signal reception and energy harvesting at the relay and is divided into two subphases. As in [15], in the first subphase, all the received signal power is used for energy harvesting. This subphase has duration ρT, where ρ is the TS ratio, 0≤ρ≤1. In the second subphase, all the received signal power is used for information decoding, and the duration is $\left(1-\rho \right)\frac{T}{2}$. *A*, *B*, and *J* send their signals $x_{A}$, $x_{B}$, and $x_{J}$ with $E[|x_{A}{|}^{2}]=E[|x_{B}{|}^{2}]=E[|x_{J}{|}^{2}=1$ and transmit powers $P_{A}$, $P_{B}$, and $P_{J}$, respectively, to *R*. The relay depends solely on the energy harvested from the user and jamming signals in the first subphase to amplify and forward the signals received from the users in the second subphase. The EH signal during the first subphase is

(2) $$y_{Re}=\sqrt{P_{A}}h_{AR}x_{A}+\sqrt{P_{B}}h_{BR}x_{B}+\sqrt{P_{J}}h_{JR}x_{J}.$$

The noise at the relay, $n_{R}$, is neglected because it is much less than the other terms in (2) [15]. The harvested energy is

(3) $$E_{H}=\rho T\zeta \left(P_{A}|h_{AR}{|}^{2}+P_{B}|h_{BR}{|}^{2}+P_{J}{\left|h_{JR}\right|}^{2}\right),$$

where ζ,0<ζ≤1, is the energy conversion efficiency. In the second phase, the relay transmit power is

(4) $$P_{R}=\frac{E_{H}}{(1-\rho )T/2}=\frac{2\rho \zeta E_{R}}{1-\rho },$$

where $E_{R}=P_{A}|h_{AR}{|}^{2}+P_{B}|h_{BR}{|}^{2}+P_{J}{\left|h_{JR}\right|}^{2}$. The information retrieval part of the received signal during the second subphase is

(5) $$\begin{array}{cc} y_{Ri} & =\sqrt{P_{A}}h_{AR}x_{A}+\sqrt{P_{B}}h_{BR}x_{B}\\ & +\sqrt{P_{J}}h_{JR}x_{J}+n_{R}. \end{array}$$

The jamming signal term $\sqrt{P_{J}}h_{JR}x_{J}$ at the relay can be cancelled from $y_{Ri}$ as in [66,67] as *A* and *B* are assumed to have a priori information of the jammer signal. Further, the jammer is located close to the relay and farther from *A* and *B*, so the jamming signal at *A* and *B* is negligible. Information regarding the jamming signal is securely shared among the jammer, relay, and users before cooperative jamming begins. However, the jamming signal may not be perfectly cancelled at the relay, which is the assumption here. A cancellation factor Φ, 0≤Φ≤1, is used to indicate the fraction of the jamming signal that is not cancelled. This fraction, $\Phi \times P_{J}$, is amplified and forwarded to *A* and *B* by the relay. The jamming signal is perfectly cancelled if Φ=0, and there is no cancellation if Φ=1. The value of Φ depends on the circuitry of the relay receiver and the CSI at *R*.

The information retrieval signal with imperfect jamming cancellation is

(6) $$\begin{array}{cc} y_{R} & =\sqrt{P_{A}}h_{AR}x_{A}+\sqrt{P_{B}}h_{BR}x_{B}\\ & +\Phi \sqrt{P_{J}}h_{JR}x_{J}+n_{R}. \end{array}$$

### 2.1. First Phase

During the first phase, the signal received at *E* is

(7) $$\begin{array}{cc} y_{E}^{\left(1\right)}= & \sqrt{P_{A}}\left({\hat{h}}_{AE}+e_{E}\right)x_{A}+\sqrt{P_{B}}\left({\hat{h}}_{BE}+e_{E}\right)x_{B}\\ \; & +\sqrt{P_{J}}\left({\hat{h}}_{JE}+e_{E}\right)x_{J}+n_{E}. \end{array}$$

The SNR at *E* for $x_{B}$ sent to *A* in this phase is

(8) $$\begin{array}{c} SNR_{E,A}^{\left(1\right)}\\ =\frac{P_{B}{\left|{\hat{h}}_{BE}\right|}^{2}}{P_{A}|{\hat{h}}_{AE}{|}^{2}+P_{J}{\left|{\hat{h}}_{JE}\right|}^{2}+\sigma_{e}^{2}\left(P_{A}+P_{B}+P_{J}\right)+\sigma^{2}}, \end{array}$$

and the SNR at *E* for $x_{A}$ sent to *B* is

(9) $$\begin{array}{c} SNR_{E,B}^{\left(1\right)}\\ =\frac{P_{A}{\left|{\hat{h}}_{AE}\right|}^{2}}{P_{B}|{\hat{h}}_{BE}{|}^{2}+P_{J}{\left|{\hat{h}}_{JE}\right|}^{2}+\sigma_{e}^{2}\left(P_{A}+P_{B}+P_{J}\right)+\sigma^{2}}. \end{array}$$

The eavesdropper does not have knowledge of the jamming signal. Therefore, $x_{J}$ is treated as additional noise, which reduces the received SNR at *E*.

### 2.2. Second Phase

During the second phase, the relay amplifies the received signal and forwards this to the users using the harvested energy. Thus, the relay transmits the signal:

(10) $$\begin{array}{cc} x_{R}= & \frac{\sqrt{P_{R}}}{\sqrt{P_{A}|h_{AR}{|}^{2}+P_{B}|h_{BR}{|}^{2}+P_{J}{\left|h_{JR}\right|}^{2}+\sigma^{2}}}\;y_{R} \end{array}$$

(11) $$\begin{array}{cc} = & \sqrt{\frac{P_{R}}{E_{R}+\sigma^{2}}}\;y_{R}, \end{array}$$

where $\sqrt{\frac{P_{R}}{E_{R}+\sigma^{2}}}$ is the relay amplifier gain. The received signal at *A* in this phase is

(12) $$\begin{array}{cc} y_{A} & =h_{AR}x_{R}+n_{A}\\ \; & =\underset{information\;signal}{\underbrace{\frac{\sqrt{P_{R}P_{B}}h_{AR}h_{BR}}{\sqrt{E_{R}+\sigma^{2}}}x_{B}}}+\underset{information\;signal}{\underbrace{\frac{\sqrt{P_{R}P_{A}}{\left|h_{AR}\right|}^{2}}{\sqrt{E_{R}+\sigma^{2}}}x_{A}}}\\ \; & +\underset{effective\;noise}{\underbrace{\Phi \frac{\sqrt{P_{R}P_{J}}h_{AR}h_{JR}}{\sqrt{E_{R}+\sigma^{2}}}x_{J}+\frac{\sqrt{P_{R}}h_{AR}n_{R}}{\sqrt{E_{R}+\sigma^{2}}}+n_{A}}}, \end{array}$$

and the received signal at *B* is

(13) $$\begin{array}{cc} y_{B} & =h_{BR}x_{R}+n_{B}\\ \; & =\underset{information\;signal}{\underbrace{\frac{\sqrt{P_{R}P_{A}}h_{AR}h_{BR}}{\sqrt{E_{R}+\sigma^{2}}}x_{A}}}+\underset{information\;signal}{\underbrace{\frac{\sqrt{P_{R}P_{B}}{\left|h_{BR}\right|}^{2}}{\sqrt{E_{R}+\sigma^{2}}}x_{B}}}\\ \; & +\underset{effective\;noise}{\underbrace{\Phi \frac{\sqrt{P_{R}P_{J}}h_{BR}h_{JR}}{\sqrt{E_{R}+\sigma^{2}}}x_{J}+\frac{\sqrt{P_{R}}h_{BR}n_{R}}{\sqrt{E_{R}+\sigma^{2}}}+n_{B}}}. \end{array}$$

*A* and *B* cancel their own signals since self-interference cancellation can be assumed [68,69]. Let

(14) $$\begin{array}{cc} \gamma_{A} & =\frac{P_{A}{\left|h_{AR}\right|}^{2}}{\sigma^{2}}, \end{array}$$

(15) $$\begin{array}{cc} \gamma_{B} & =\frac{P_{B}{\left|h_{BR}\right|}^{2}}{\sigma^{2}}, \end{array}$$

(16) $$\begin{array}{cc} \gamma_{J} & =\frac{P_{J}{\left|h_{JR}\right|}^{2}}{\sigma^{2}}, \end{array}$$

(17) $$\begin{array}{cc} \bar{\gamma } & =\gamma_{A}+\gamma_{B}+\gamma_{J}=\frac{E_{R}}{\sigma^{2}}. \end{array}$$

The SNR at *A* is then

(18) $$SNR_{A}=\frac{2\rho \zeta \bar{\gamma }\gamma_{B}{\left|h_{AR}\right|}^{2}}{2\rho \zeta \bar{\gamma }{\left|h_{AR}\right|}^{2}\left(\Phi^{2}\gamma_{J}+1\right)+\tilde{\rho }\left(\bar{\gamma }+1\right)},$$

where $\tilde{\rho }=1-\rho$ and the achievable rate at *A* is [70]

(19) $$R_{A}=\left(1-\rho \right)\frac{T}{2}\log_{2}\left(1+SNR_{A}\right).$$

The SNR at *B* is

(20) $$\begin{array}{c} SNR_{B}=\frac{2\rho \zeta \bar{\gamma }\gamma_{A}{\left|h_{BR}\right|}^{2}}{2\rho \zeta \bar{\gamma }{\left|h_{BR}\right|}^{2}\left(\Phi^{2}\gamma_{J}+1\right)+\tilde{\rho }\left(\bar{\gamma }+1\right)}, \end{array}$$

and the achievable rate at *B* is

(21) $$R_{B}=\left(1-\rho \right)\frac{T}{2}\log_{2}\left(1+SNR_{B}\right).$$

The signal received at *E* during the second phase is

(22) $$\begin{array}{cc} y_{E}^{\left(2\right)} & =h_{RE}x_{R}+n_{E},\\ \; & =\underset{information\;signal}{\underbrace{\frac{\sqrt{P_{R}P_{A}}h_{AR}h_{RE}}{\sqrt{E_{R}+\sigma^{2}}}x_{A}}}+\underset{information\;signal}{\underbrace{\frac{\sqrt{P_{R}P_{B}}h_{BR}h_{RE}}{\sqrt{E_{R}+\sigma^{2}}}x_{B}}} \end{array}$$

(23) $$\begin{array}{cc} \; & +\underset{effective\;noise}{\underbrace{\Phi \frac{\sqrt{P_{R}P_{J}}h_{JR}h_{RE}}{\sqrt{E_{R}+\sigma^{2}}}x_{J}+\frac{\sqrt{P_{R}}h_{RE}n_{R}}{\sqrt{E_{R}+\sigma^{2}}}+n_{E}}}, \end{array}$$

where $h_{RE}={\hat{h}}_{RE}+e_{E}$. The SNR at *E* for $x_{B}$ sent to *A* during the second phase is

(24) $$\begin{array}{ccc} SNR_{E,A}^{\left(2\right)} & = & \frac{2\rho \zeta \bar{\gamma }\gamma_{B}{\left|{\hat{h}}_{RE}\right|}^{2}}{2\rho \zeta \bar{\gamma }\left[\right|{\hat{h}}_{RE}{|}^{2}\left(\gamma_{A}+\Phi^{2}\gamma_{J}+1\right)} \end{array}$$

(25) $$\begin{array}{ccc} \; & \; & +\sigma_{e}^{2}\left(\gamma_{A}+\gamma_{B}+\Phi^{2}\gamma_{J}+1\right)]+\tilde{\rho }\left(\bar{\gamma }+1\right), \end{array}$$

and the SNR at *E* for $x_{A}$ sent to *B* during the second phase is

(26) $$\begin{array}{ccc} SNR_{E,B}^{\left(2\right)} & = & \frac{2\rho \zeta \bar{\gamma }\gamma_{A}{\left|{\hat{h}}_{RE}\right|}^{2}}{2\rho \zeta \bar{\gamma }\left[\right|{\hat{h}}_{RE}{|}^{2}\left(\gamma_{B}+\Phi^{2}\gamma_{J}+1\right)} \end{array}$$

(27) $$\begin{array}{ccc} \; & \; & +\sigma_{e}^{2}\left(\gamma_{A}+\gamma_{B}+\Phi^{2}\gamma_{J}+1\right)]+\tilde{\rho }\left(\bar{\gamma }+1\right). \end{array}$$

The achievable rate at *E* during both phases is then

(28) $$R_{E,i}=\left\{\begin{array}{c} \left(1-\rho \right)\frac{T}{2}\log_{2}\left(1+SNR_{E,i}^{\left(1\right)}+SNR_{E,i}^{\left(2\right)}\right),\\ \;\;\mathrm{MRC}\;\mathrm{at}\;E\\ \left(1-\rho \right)\frac{T}{2}\log_{2}\left(1+\max\left(SNR_{E,i}^{\left(1\right)}+SNR_{E,i}^{\left(2\right)}\right)\right),\\ \;\mathrm{SC}\;\mathrm{at}\;E. \end{array}\right.$$

## 3. Secrecy Capacity Analysis

The secrecy capacity in the presence of an eavesdropper is the difference between the secrecy capacity of the link between the users and the secrecy capacity of the wiretap link [70]. The total transmit power in this network is limited by the total power constraint $P_{T}$, where $P_{A}+P_{B}+P_{J}\leq P_{T}$. The goal is to determine the time switching ratio and transmit power of *A*, *B*, and *J* to maximize the secrecy capacity at *A* and *B* under this constraint. The secrecy capacity at *A* is $C_{S,A}={\left[R_{A}-R_{E,A}\right]}^{+}$ and at *B* is $C_{S,B}={\left[R_{B}-R_{E,B}\right]}^{+}$ [71], where ${\left[x\right]}^{+}=\max\left(0,x\right)$. The secrecy capacity at user *i*, $i\in \left\{A,B\right\}$, is then

(29) $$C_{S,i}=\left\{\begin{array}{c} \left(1-\rho \right)\frac{T}{2}\log_{2}\left(\frac{1+SNR_{i}}{1+SNR_{E,i}^{\left(1\right)}+SNR_{E,i}^{\left(2\right)}}\right),\\ \;\mathrm{MRC}\;\mathrm{at}\;E\\ \left(1-\rho \right)\frac{T}{2}\log_{2}\left(\frac{1+SNR_{i}}{1+\max(SNR_{E,i}^{\left(1\right)}+SNR_{E,i}^{\left(2\right)})}\right),\\ \;\mathrm{SC}\;\mathrm{at}\;E. \end{array}\right.$$

The secrecy capacity is

(30) $$\begin{array}{ccc} C_{S} & = & C_{S,A}+C_{S,B}, \end{array}$$

(31) $$\begin{array}{ccc} \; & = & {\left[R_{A}-R_{E,A}\right]}^{+}+{\left[R_{B}-R_{E,B}\right]}^{+}, \end{array}$$

and the corresponding optimization problem is formulated as

$$\begin{array}{cc} \max_{\rho ,\tilde{\rho },P_{A},P_{B},P_{J}}\;C_{S}\\ P_{A}+P_{B}+P_{J} & \leq \;P_{T}\\ \rho +\tilde{\rho } & \leq \;1\\ \rho ,\tilde{\rho },P_{A},P_{B},P_{J} & \geq 0 \end{array}$$

### 3.1. MRC at the Eavesdropper

In this subsection, the secrecy capacity of the communication system is investigated with imperfect channel estimation at the eavesdropper. The eavesdropper employs MRC to combine the signals from the direct and relay links in both transmission phases. The achievable rates at *E* for $x_{B}$ sent to A and $x_{A}$ sent to *B*, $R_{E,A}$, and $R_{E,B}$, respectively, are defined in (28). $C_{S,A}$ is obtained by substituting $SNR_{A}$, $SNR_{E,A}^{\left(1\right)}$, and $SNR_{E,A}^{\left(2\right)}$ given by (18), (8), and (25), respectively, in (29) with i=A. $C_{S,B}$ is obtained by substituting $SNR_{B}$, $SNR_{E,B}^{\left(1\right)}$, and $SNR_{E,B}^{\left(2\right)}$ given by (20), (9), and (27), respectively, in (29) with i=B. From (31), there are four cases to consider to maximize the secrecy capacity as given below.

#### 3.1.1. Case I: $C_{S,A}\geq 0$ and $C_{S,B}\geq 0$

In this case, the secrecy capacity is

(32) $$\begin{array}{cc} C_{S}= & \left(R_{A}-R_{E,A}\right)+\left(R_{B}-R_{E,B}\right)\\ = & \frac{T}{2}\log_{2}\left(\frac{w_{I}^{MRC}}{z_{I}^{MRC}}\right), \end{array}$$

where ${\left(.\right)}_{I}^{MRC}$ denotes the first case with MRC at the eavesdropper:

(33) $$\begin{array}{cc} \; & w_{I}^{MRC}=\\ \; & \left(2\rho \zeta \bar{\gamma }\gamma_{B}|h_{AR}{|}^{2}+2\rho \zeta \bar{\gamma }|h_{AR}{|}^{2}\left(\Phi^{2}\gamma_{J}+1\right)+\tilde{\rho }\left(\bar{\gamma }+1\right)\right)\\ \; & \left(P_{A}|{\hat{h}}_{AE}{|}^{2}+P_{J}|{\hat{h}}_{JE}{|}^{2}+\sigma_{e}^{2}\left(P_{A}+P_{B}+P_{J}\right)+\sigma^{2}\right)\\ \; & (2\rho \zeta \bar{\gamma }\left[\right|{\hat{h}}_{RE}{|}^{2}\left(\gamma_{A}+\Phi^{2}\gamma_{J}+1\right)+\\ \; & \;\sigma_{e}^{2}\left(\gamma_{A}+\gamma_{B}+\Phi^{2}\gamma_{J}+1\right)]+\tilde{\rho }\left(\bar{\gamma }+1\right))\\ \; & (2\rho \zeta \bar{\gamma }\gamma_{A}|h_{BR}{|}^{2})+(2\rho \zeta \bar{\gamma }|h_{BR}{|}^{2}\left(\Phi^{2}\gamma_{J}+1\right)+\tilde{\rho }\left(\bar{\gamma }+1\right))\\ \; & \left(P_{B}|{\hat{h}}_{BE}{|}^{2}+P_{J}|{\hat{h}}_{JE}{|}^{2}+\sigma_{e}^{2}\left(P_{A}+P_{B}+P_{J}\right)+\sigma^{2}\right)\\ \; & (2\rho \zeta \bar{\gamma }\left[\right|{\hat{h}}_{RE}{|}^{2}\left(\gamma_{B}+\Phi^{2}\gamma_{J}+1\right)+\sigma_{e}^{2}\left(\gamma_{A}+\gamma_{B}+\Phi^{2}\gamma_{J}+1\right)] \end{array}$$

(34) $$\begin{array}{cc} \; & \;+\tilde{\rho }\left(\bar{\gamma }+1\right)), \end{array}$$

and

(35) $$\begin{array}{cc} \; & z_{I}^{MRC}=\\ \; & \left(2\rho \zeta \bar{\gamma }|h_{AR}{|}^{2}\left(\Phi^{2}\gamma_{J}+1\right)+\tilde{\rho }\left(\bar{\gamma }+1\right)\right)\\ \; & \left(2\rho \zeta \bar{\gamma }|h_{BR}{|}^{2}\left(\Phi^{2}\gamma_{J}+1\right)+\tilde{\rho }\left(\bar{\gamma }+1\right)\right)\\ \; & [((P_{B}|{\hat{h}}_{BE}{|}^{2})+(P_{A}|{\hat{h}}_{AE}{|}^{2}+P_{J}{\left|{\hat{h}}_{JE}\right|}^{2}\\ \; & \;+\sigma_{e}^{2}\left(P_{A}+P_{B}+P_{J}\right)+\sigma^{2}))(2\rho \zeta \bar{\gamma }\left[\right|{\hat{h}}_{RE}{|}^{2}\left(\gamma_{A}+\Phi^{2}\gamma_{J}+1\right)\\ & \;+\sigma_{e}^{2}\left(\gamma_{A}+\gamma_{B}+\Phi^{2}\gamma_{J}+1\right)]\\ \; & \;+\tilde{\rho }\left(\bar{\gamma }+1\right))+(P_{A}|{\hat{h}}_{AE}{|}^{2}+P_{J}{\left|{\hat{h}}_{JE}\right|}^{2}+\sigma_{e}^{2}\left(P_{A}+P_{B}+P_{J}\right)\\ \; & \;+\sigma^{2})(2\rho \zeta \bar{\gamma }\gamma_{B}|{\hat{h}}_{RE}{|}^{2})]\\ \; & [((P_{A}|{\hat{h}}_{AE}{|}^{2})+(P_{B}|{\hat{h}}_{BE}{|}^{2}+P_{J}{\left|{\hat{h}}_{JE}\right|}^{2}+\sigma_{e}^{2}\left(P_{A}+P_{B}+P_{J}\right)\\ \; & \;+\sigma^{2}))(2\rho \zeta \bar{\gamma }\left[\right|{\hat{h}}_{RE}{|}^{2}\left(\gamma_{B}+\Phi^{2}\gamma_{J}+1\right)+\sigma_{e}^{2}(\gamma_{A}+\gamma_{B}+\Phi^{2}\gamma_{J}\\ \; & \;+1)]+\tilde{\rho }\left(\bar{\gamma }+1\right))+(P_{B}|{\hat{h}}_{BE}{|}^{2}+P_{J}|{\hat{h}}_{JE}{|}^{2}+\sigma_{e}^{2}(P_{A}+P_{B}\\ \; & \;+P_{J})+\sigma^{2})(2\rho \zeta \bar{\gamma }\gamma_{A}|{\hat{h}}_{RE}{|}^{2})]. \end{array}$$

#### 3.1.2. Case II: $C_{S,A}\geq 0$ and $C_{S,B}\leq 0$

In this case, $C_{S,B}=0$ since the SNR at the eavesdropper is higher than that at *B*. The secrecy capacity is then

(36) $$\begin{array}{cc} C_{S}= & \left(R_{A}-R_{E,A}\right)\\ = & \frac{T}{2}\log_{2}\left(\frac{w_{II}^{MRC}}{z_{II}^{MRC}}\right), \end{array}$$

where ${\left(.\right)}_{II}^{MRC}$ denotes the second case with MRC at the eavesdropper:

(37) $$\begin{array}{cc} \; & w_{II}^{MRC}=\\ \; & ((2\rho \zeta \bar{\gamma }\gamma_{B}|h_{AR}{|}^{2})+(2\rho \zeta \bar{\gamma }|h_{AR}{|}^{2}\left(\Phi^{2}\gamma_{J}+1\right)+\tilde{\rho }\left(\bar{\gamma }+1\right)))\\ \; & \left(P_{A}|{\hat{h}}_{AE}{|}^{2}+P_{J}|{\hat{h}}_{JE}{|}^{2}+\sigma_{e}^{2}\left(P_{A}+P_{B}+P_{J}\right)+\sigma^{2}\right)\\ \; & (2\rho \zeta \bar{\gamma }\left[\right|{\hat{h}}_{RE}{|}^{2}\left(\gamma_{A}+\Phi^{2}\gamma_{J}+1\right)+\sigma_{e}^{2}(\gamma_{A}+\gamma_{B}+\Phi^{2}\gamma_{J}\\ & \;+1)]+\tilde{\rho }\left(\bar{\gamma }+1\right)), \end{array}$$

and

(38) $$\begin{array}{cc} \; & z_{II}^{MRC}=\\ \; & \left(2\rho \zeta \bar{\gamma }|h_{AR}{|}^{2}\left(\Phi^{2}\gamma_{J}+1\right)+\tilde{\rho }\left(\bar{\gamma }+1\right)\right)\\ \; & [((P_{B}|{\hat{h}}_{BE}{|}^{2})+(P_{B}|{\hat{h}}_{BE}{|}^{2}+P_{J}{\left|{\hat{h}}_{JE}\right|}^{2}\\ \; & \;+\sigma_{e}^{2}\left(P_{A}+P_{B}+P_{J}\right)+\sigma^{2}))(2\rho \zeta \bar{\gamma }\left[\right|{\hat{h}}_{RE}{|}^{2}(\gamma_{A}\\ \; & \;+\Phi^{2}\gamma_{J}+1)+\sigma_{e}^{2}\left(\gamma_{A}+\gamma_{B}+\Phi^{2}\gamma_{J}+1\right)]\\ \; & \;+\tilde{\rho }\left(\bar{\gamma }+1\right))+((P_{A}|{\hat{h}}_{AE}{|}^{2}+P_{J}|{\hat{h}}_{JE}{|}^{2}+\sigma_{e}^{2}(P_{A}+P_{B}\\ \; & \;+P_{J})+\sigma^{2})*(2\rho \zeta \bar{\gamma }\gamma_{B}|{\hat{h}}_{RE}{|}^{2}))]. \end{array}$$

#### 3.1.3. Case III: $C_{S,A}\leq 0$ and $C_{S,B}\geq 0$

In this case, $C_{S,A}=0$ since the SNR at the eavesdropper is higher than that at *A*. The secrecy capacity is then

(39) $$\begin{array}{cc} C_{S}= & \left(R_{B}-R_{E,B}\right)\\ = & \frac{T}{2}\log_{2}\left(\frac{w_{III}^{MRC}}{z_{III}^{MRC}}\right), \end{array}$$

where ${\left(.\right)}_{III}^{MRC}$ denotes the third case with MRC at the eavesdropper:

(40) $$\begin{array}{cc} \; & w_{III}^{MRC}=\\ \; & ((2\rho \zeta \bar{\gamma }\gamma_{A}|h_{BR}{|}^{2})+(2\rho \zeta \bar{\gamma }|h_{BR}{|}^{2}\left(\Phi^{2}\gamma_{J}+1\right)+\tilde{\rho }\left(\bar{\gamma }+1\right)))\\ \; & \left(P_{B}|{\hat{h}}_{BE}{|}^{2}+P_{J}|{\hat{h}}_{JE}{|}^{2}+\sigma_{e}^{2}\left(P_{A}+P_{B}+P_{J}\right)+\sigma^{2}\right)\\ \; & (2\rho \zeta \bar{\gamma }\left[\right|{\hat{h}}_{RE}{|}^{2}\left(\gamma_{B}+\Phi^{2}\gamma_{J}+1\right)+\sigma_{e}^{2}\left(\gamma_{A}+\gamma_{B}+\Phi^{2}\gamma_{J}+1\right)]\\ \; & \;+\tilde{\rho }\left(\bar{\gamma }+1\right)), \end{array}$$

and

(41) $$\begin{array}{cc} \; & z_{III}^{MRC}=\\ \; & \left(2\rho \zeta \bar{\gamma }|h_{BR}{|}^{2}\left(\Phi^{2}\gamma_{J}+1\right)+\tilde{\rho }\left(\bar{\gamma }+1\right)\right)\\ \; & [((P_{A}|{\hat{h}}_{AE}{|}^{2})+(P_{B}|{\hat{h}}_{BE}{|}^{2}+P_{J}{\left|{\hat{h}}_{JE}\right|}^{2}+\sigma_{e}^{2}\left(P_{A}+P_{B}+P_{J}\right)\\ \; & \;+\sigma^{2}))(2\rho \zeta \bar{\gamma }\left[\right|{\hat{h}}_{RE}{|}^{2}\left(\gamma_{B}+\Phi^{2}\gamma_{J}+1\right)\\ \; & \;+\sigma_{e}^{2}\left(\gamma_{A}+\gamma_{B}+\Phi^{2}\gamma_{J}+1\right)]+\tilde{\rho }\left(\bar{\gamma }+1\right))(P_{B}{\left|{\hat{h}}_{BE}\right|}^{2}\\ \; & \;+P_{J}|{\hat{h}}_{JE}{|}^{2}+\sigma_{e}^{2}\left(P_{A}+P_{B}+P_{J}\right)+\sigma^{2})(2\rho \zeta \bar{\gamma }\gamma_{A}|{\hat{h}}_{RE}{|}^{2})]. \end{array}$$

#### 3.1.4. Case IV: $C_{S,A}\leq 0$ and $C_{S,B}\leq 0$

In this case, the secrecy capacity is $C_{S}=0$ because the secrecy capacity of the wiretapped links is higher than the secrecy capacity at *A* and *B*.

### 3.2. SC at the Eavesdropper

In this subsection, the secrecy capacity of the communication system is investigated with imperfect channel estimation at the eavesdropper. The eavesdropper employs SC so the link (direct or relay) with the maximum SNR is selected. Based on $SNR_{E,A}^{\left(1\right)}$, $SNR_{E,A}^{\left(2\right)}$, $SNR_{E,B}^{\left(1\right)}$, and $SNR_{E,B}^{\left(2\right)}$ given by (8), (25), (9), and (27), respectively, the following four cases can be considered.

#### 3.2.1. Case I: $SNR_{E,A}^{\left(1\right)}\geq SNR_{E,A}^{\left(2\right)}$ and $SNR_{E,B}^{\left(1\right)}\geq SNR_{E,B}^{\left(2\right)}$

In this case, the secrecy capacity is

(42) $$\begin{array}{cc} C_{S}= & C_{S,A}+C_{S,B}\\ = & \frac{T}{2}\log_{2}\left(\frac{1+SNR_{A}}{1+SNR_{E,A}^{\left(1\right)}}\right)+\frac{T}{2}\log_{2}\left(\frac{1+SNR_{B}}{1+SNR_{E,B}^{\left(1\right)}}\right),\\ = & \frac{T}{2}\log_{2}\left(\frac{w_{I,A}^{SC}}{z_{I,A}^{SC}}\right)+\frac{T}{2}\log_{2}\left(\frac{w_{I,B}^{SC}}{z_{I,B}^{SC}}\right),\\ = & \frac{T}{2}\log_{2}\left(\frac{w_{I}^{SC}}{z_{I}^{SC}}\right), \end{array}$$

where

(43) $$\begin{array}{c} \begin{array}{cc} \; & w_{I,A}^{SC}=\\ \; & ((2\rho \zeta \bar{\gamma }\gamma_{B}|h_{AR}{|}^{2})+(2\rho \zeta \bar{\gamma }|h_{AR}{|}^{2}\left(\Phi^{2}\gamma_{J}+1\right)+\tilde{\rho }\left(\bar{\gamma }+1\right)))\\ \; & (P_{A}|{\hat{h}}_{AE}{|}^{2}+P_{J}|{\hat{h}}_{JE}{|}^{2}+\sigma_{e}^{2}\left(P_{A}+P_{B}+P_{J}\right)+\sigma^{2}), \end{array} \end{array}$$

(44) $$\begin{array}{c} \begin{array}{cc} \; & w_{I,B}^{SC}=\\ \; & ((2\rho \zeta \bar{\gamma }\gamma_{A}|h_{BR}{|}^{2})+(2\rho \zeta \bar{\gamma }|h_{BR}{|}^{2}\left(\Phi^{2}\gamma_{J}+1\right)+\tilde{\rho }\left(\bar{\gamma }+1\right)))\\ \; & (P_{B}|{\hat{h}}_{BE}{|}^{2}+P_{J}|{\hat{h}}_{JE}{|}^{2}+\sigma_{e}^{2}\left(P_{A}+P_{B}+P_{J}\right)+\sigma^{2}), \end{array} \end{array}$$

(45) $$\begin{array}{c} \begin{array}{cc} \; & z_{I,A}^{SC}=\\ \; & ((P_{B}|{\hat{h}}_{BE}{|}^{2})+(P_{A}|{\hat{h}}_{AE}{|}^{2}+P_{J}{\left|{\hat{h}}_{JE}\right|}^{2}\\ \; & \;+\sigma_{e}^{2}\left(P_{A}+P_{B}+P_{J}\right)+\sigma^{2})\\ \; & (2\rho \zeta \bar{\gamma }|h_{AR}{|}^{2}\left(\Phi^{2}\gamma_{J}+1\right)+\tilde{\rho }\left(\bar{\gamma }+1\right)), \end{array} \end{array}$$

(46) $$\begin{array}{c} \begin{array}{cc} \; & z_{I,B}^{SC}=\\ \; & ((P_{A}|{\hat{h}}_{AE}{|}^{2})+(P_{B}|{\hat{h}}_{BE}{|}^{2}+P_{J}{\left|{\hat{h}}_{JE}\right|}^{2}\\ \; & \;+\sigma_{e}^{2}\left(P_{A}+P_{B}+P_{J}\right)+\sigma^{2}))\\ \; & (2\rho \zeta \bar{\gamma }|h_{BR}{|}^{2}\left(\Phi^{2}\gamma_{J}+1\right)+\tilde{\rho }\left(\bar{\gamma }+1\right)), \end{array} \end{array}$$

(47) $$\frac{w_{I}^{SC}}{z_{I}^{SC}}=\left\{\begin{array}{cc} \frac{w_{I,A}^{SC}w_{I,B}^{SC}}{z_{I,A}^{SC}z_{I,B}^{SC}}, & C_{S,A}\geq 0\;and\;C_{S,B}\geq 0\\ \frac{w_{I,A}^{SC}}{z_{I,A}^{SC}}, & C_{S,A}\geq 0\;and\;C_{S,B}<0\\ \frac{w_{I,B}^{SC}}{z_{I,B}^{SC}}, & C_{S,A}<0\;and\;C_{S,B}\geq 0\\ 0, & C_{S,A}<0\;and\;C_{S,B}<0, \end{array}\right.$$

and ${\left(.\right)}_{I}^{SC}$ denotes the first case with SC at the eavesdropper.

#### 3.2.2. Case II: $SNR_{E,A}^{\left(1\right)}\geq SNR_{E,A}^{\left(2\right)}$ and $SNR_{E,B}^{\left(1\right)}\leq SNR_{E,B}^{\left(2\right)}$

In this case, the secrecy capacity is

(48) $$\begin{array}{cc} C_{S}= & C_{S,A}+C_{S,B}\\ = & \frac{T}{2}\log_{2}\left(\frac{1+SNR_{A}}{1+SNR_{E,A}^{\left(1\right)}}\right)+\frac{T}{2}\log_{2}\left(\frac{1+SNR_{B}}{1+SNR_{E,B}^{\left(2\right)}}\right),\\ = & \frac{T}{2}\log_{2}\left(\frac{w_{II,A}^{SC}}{z_{II,A}^{SC}}\right)+\frac{T}{2}\log_{2}\left(\frac{w_{II,B}^{SC}}{z_{II,B}^{SC}}\right),\\ = & \frac{T}{2}\log_{2}\left(\frac{w_{II}^{SC}}{z_{II}^{SC}}\right), \end{array}$$

where

(49) $$\begin{array}{c} \begin{array}{cc} \; & w_{II,A}^{SC}=\\ \; & ((2\rho \zeta \bar{\gamma }\gamma_{B}|h_{AR}{|}^{2})+(2\rho \zeta \bar{\gamma }|h_{AR}{|}^{2}\left(\Phi^{2}\gamma_{J}+1\right)+\tilde{\rho }\left(\bar{\gamma }+1\right)))\\ \; & (P_{A}|{\hat{h}}_{AE}{|}^{2}+P_{J}|{\hat{h}}_{JE}{|}^{2}+\sigma_{e}^{2}\left(P_{A}+P_{B}+P_{J}\right)+\sigma^{2}), \end{array} \end{array}$$

(50) $$\begin{array}{c} \begin{array}{cc} \; & w_{II,B}^{SC}=\\ \; & ((2\rho \zeta \bar{\gamma }\gamma_{A}|h_{BR}{|}^{2})+(2\rho \zeta \bar{\gamma }|h_{BR}{|}^{2}\left(\Phi^{2}\gamma_{J}+1\right)+\tilde{\rho }\left(\bar{\gamma }+1\right)))\\ \; & (2\rho \zeta \bar{\gamma }\left[\right|{\hat{h}}_{RE}{|}^{2}\left(\gamma_{B}+\Phi^{2}\gamma_{J}+1\right)+\sigma_{e}^{2}\left(\gamma_{A}+\gamma_{B}+\Phi^{2}\gamma_{J}+1\right)]\\ \; & \;+\tilde{\rho }\left(\bar{\gamma }+1\right)), \end{array} \end{array}$$

(51) $$\begin{array}{c} \begin{array}{cc} \; & z_{II,A}^{SC}=\\ \; & ((P_{B}|{\hat{h}}_{BE}{|}^{2})+(P_{A}|{\hat{h}}_{AE}{|}^{2}+P_{J}{\left|{\hat{h}}_{JE}\right|}^{2}+\sigma_{e}^{2}\left(P_{A}+P_{B}+P_{J}\right)\\ \; & \;+\sigma^{2})(2\rho \zeta \bar{\gamma }|h_{AR}{|}^{2}\left(\Phi^{2}\gamma_{J}+1\right)+\tilde{\rho }\left(\bar{\gamma }+1\right)), \end{array} \end{array}$$

(52) $$\begin{array}{c} \begin{array}{cc} \; & z_{II,B}^{SC}=\\ \; & ((2\rho \zeta \bar{\gamma }\gamma_{A}|{\hat{h}}_{RE}{|}^{2})+(2\rho \zeta \bar{\gamma }\left[\right|{\hat{h}}_{RE}{|}^{2}\left(\gamma_{B}+\Phi^{2}\gamma_{J}+1\right)\\ \; & \;+\sigma_{e}^{2}\left(\gamma_{A}+\gamma_{B}+\Phi^{2}\gamma_{J}+1\right)]+\tilde{\rho }\left(\bar{\gamma }+1\right)))\\ \; & \;(2\rho \zeta \bar{\gamma }|h_{BR}{|}^{2}\left(\Phi^{2}\gamma_{J}+1\right)+\tilde{\rho }\left(\bar{\gamma }+1\right)), \end{array} \end{array}$$

(53) $$\frac{w_{II}^{SC}}{z_{II}^{SC}}=\left\{\begin{array}{cc} \frac{w_{II,A}^{SC}w_{II,B}^{SC}}{z_{II,A}^{SC}z_{II,B}^{SC}}, & C_{S,A}\geq 0\;and\;C_{S,B}\geq 0\\ \frac{w_{II,A}^{SC}}{z_{II,A}^{SC}}, & C_{S,A}\geq 0\;and\;C_{S,B}<0\\ \frac{w_{II,B}^{SC}}{z_{II,B}^{SC}}, & C_{S,A}<0\;and\;C_{S,B}\geq 0\\ 0, & C_{S,A}<0\;and\;C_{S,B}<0, \end{array}\right.$$

and ${\left(.\right)}_{II}^{SC}$ denotes the second case with SC at the eavesdropper.

#### 3.2.3. Case III: $SNR_{E,A}^{\left(1\right)}\leq SNR_{E,A}^{\left(2\right)}$ and $SNR_{E,B}^{\left(1\right)}\geq SNR_{E,B}^{\left(2\right)}$

In this case, the secrecy capacity is

(54) $$\begin{array}{cc} C_{S}= & C_{S,A}+C_{S,B}\\ = & \frac{T}{2}\log_{2}\left(\frac{1+SNR_{A}}{1+SNR_{E,A}^{\left(2\right)}}\right)+\frac{T}{2}\log_{2}\left(\frac{1+SNR_{B}}{1+SNR_{E,B}^{\left(1\right)}}\right),\\ = & \frac{T}{2}\log_{2}\left(\frac{w_{III,A}^{SC}}{z_{III,A}^{SC}}\right)+\frac{T}{2}\log_{2}\left(\frac{w_{III,B}^{SC}}{z_{III,B}^{SC}}\right),\\ = & \frac{T}{2}\log_{2}\left(\frac{w_{III}^{SC}}{z_{III}^{SC}}\right), \end{array}$$

where

(55) $$\begin{array}{c} \begin{array}{cc} \; & w_{III,A}^{SC}=\\ \; & ((2\rho \zeta \bar{\gamma }\gamma_{B}|h_{AR}{|}^{2})+(2\rho \zeta \bar{\gamma }|h_{AR}{|}^{2}\left(\Phi^{2}\gamma_{J}+1\right)+\tilde{\rho }\left(\bar{\gamma }+1\right)))\\ \; & (2\rho \zeta \bar{\gamma }\left[\right|{\hat{h}}_{RE}{|}^{2}\left(\gamma_{A}+\Phi^{2}\gamma_{J}+1\right)+\sigma_{e}^{2}\left(\gamma_{A}+\gamma_{B}+\Phi^{2}\gamma_{J}+1\right)]\\ \; & \;+\tilde{\rho }\left(\bar{\gamma }+1\right)), \end{array} \end{array}$$

(56) $$\begin{array}{c} \begin{array}{cc} \; & w_{III,B}^{SC}=\\ \; & ((2\rho \zeta \bar{\gamma }\gamma_{A}|h_{BR}{|}^{2})+(2\rho \zeta \bar{\gamma }|h_{BR}{|}^{2}\left(\Phi^{2}\gamma_{J}+1\right)+\tilde{\rho }\left(\bar{\gamma }+1\right)))\\ \; & (P_{B}|{\hat{h}}_{BE}{|}^{2}+P_{J}|{\hat{h}}_{JE}{|}^{2}+\sigma_{e}^{2}\left(P_{A}+P_{B}+P_{J}\right)+\sigma^{2}), \end{array} \end{array}$$

(57) $$\begin{array}{c} \begin{array}{cc} \; & z_{III,A}^{SC}=\\ \; & ((2\rho \zeta \bar{\gamma }\gamma_{B}|{\hat{h}}_{RE}{|}^{2})+(2\rho \zeta \bar{\gamma }\left[\right|{\hat{h}}_{RE}{|}^{2}\left(\gamma_{A}+\Phi^{2}\gamma_{J}+1\right)\\ \; & \;+\sigma_{e}^{2}\left(\gamma_{A}+\gamma_{B}+\Phi^{2}\gamma_{J}+1\right)]+\tilde{\rho }\left(\bar{\gamma }+1\right)))\\ \; & \;(2\rho \zeta \bar{\gamma }|h_{AR}{|}^{2}\left(\Phi^{2}\gamma_{J}+1\right)+\tilde{\rho }\left(\bar{\gamma }+1\right)), \end{array} \end{array}$$

(58) $$\begin{array}{c} \begin{array}{cc} \; & z_{III,B}^{SC}=\\ \; & ((P_{A}|{\hat{h}}_{AE}{|}^{2})+(P_{B}|{\hat{h}}_{BE}{|}^{2}+P_{J}{\left|{\hat{h}}_{JE}\right|}^{2}\\ \; & \;+\sigma_{e}^{2}\left(P_{A}+P_{B}+P_{J}\right)+\sigma^{2}))\\ \; & (2\rho \zeta \bar{\gamma }|h_{BR}{|}^{2}\left(\Phi^{2}\gamma_{J}+1\right)+\tilde{\rho }\left(\bar{\gamma }+1\right)), \end{array} \end{array}$$

(59) $$\frac{w_{III}^{SC}}{z_{III}^{SC}}=\left\{\begin{array}{cc} \frac{w_{III,A}^{SC}w_{III,B}^{SC}}{z_{III,A}^{SC}z_{III,B}^{SC}}, & C_{S,A}\geq 0\;and\;C_{S,B}\geq 0\\ \frac{w_{III,A}^{SC}}{z_{III,A}^{SC}}, & C_{S,A}\geq 0\;and\;C_{S,B}<0\\ \frac{w_{III,B}^{SC}}{z_{III,B}^{SC}}, & C_{S,A}<0\;and\;C_{S,B}\geq 0\\ 0, & C_{S,A}<0\;and\;C_{S,B}<0, \end{array}\right.$$

and ${\left(.\right)}_{III}^{SC}$ denotes the third case with SC at the eavesdropper.

#### 3.2.4. Case IV: $SNR_{E,A}^{\left(1\right)}\leq SNR_{E,A}^{\left(2\right)}$ and $SNR_{E,B}^{\left(1\right)}\leq SNR_{E,B}^{\left(2\right)}$

In this case, the secrecy capacity is

(60) $$\begin{array}{cc} C_{S}= & C_{S,A}+C_{S,B}\\ = & \frac{T}{2}\log_{2}\left(\frac{1+SNR_{A}}{1+SNR_{E,A}^{\left(2\right)}}\right)+\frac{T}{2}\log_{2}\left(\frac{1+SNR_{B}}{1+SNR_{E,B}^{\left(2\right)}}\right),\\ = & \frac{T}{2}\log_{2}\left(\frac{w_{IV,A}^{SC}}{z_{IV,A}^{SC}}\right)+\frac{T}{2}\log_{2}\left(\frac{w_{IV,B}^{SC}}{z_{IV,B}^{SC}}\right),\\ = & \frac{T}{2}\log_{2}\left(\frac{w_{IV}^{SC}}{z_{IV}^{SC}}\right), \end{array}$$

where

(61) $$\begin{array}{c} \begin{array}{cc} \; & w_{IV,A}^{SC}=\\ \; & ((2\rho \zeta \bar{\gamma }\gamma_{B}|h_{AR}{|}^{2})+(2\rho \zeta \bar{\gamma }|h_{AR}{|}^{2}\left(\Phi^{2}\gamma_{J}+1\right)+\tilde{\rho }\left(\bar{\gamma }+1\right)))\\ \; & (2\rho \zeta \bar{\gamma }\left[\right|{\hat{h}}_{RE}{|}^{2}\left(\gamma_{A}+\Phi^{2}\gamma_{J}+1\right)+\sigma_{e}^{2}\left(\gamma_{A}+\gamma_{B}+\Phi^{2}\gamma_{J}+1\right)]\\ \; & \;+\tilde{\rho }\left(\bar{\gamma }+1\right)), \end{array} \end{array}$$

(62) $$\begin{array}{c} \begin{array}{cc} \; & w_{IV,B}^{SC}=\\ \; & ((2\rho \zeta \bar{\gamma }\gamma_{A}|h_{BR}{|}^{2})+(2\rho \zeta \bar{\gamma }|h_{BR}{|}^{2}\left(\Phi^{2}\gamma_{J}+1\right)+\tilde{\rho }\left(\bar{\gamma }+1\right)))\\ \; & (2\rho \zeta \bar{\gamma }\left[\right|{\hat{h}}_{RE}{|}^{2}\left(\gamma_{B}+\Phi^{2}\gamma_{J}+1\right)+\sigma_{e}^{2}\left(\gamma_{A}+\gamma_{B}+\Phi^{2}\gamma_{J}+1\right)]\\ \; & \;+\tilde{\rho }\left(\bar{\gamma }+1\right)), \end{array} \end{array}$$

(63) $$\begin{array}{c} \begin{array}{cc} \; & z_{IV,A}^{SC}=\\ \; & ((2\rho \zeta \bar{\gamma }\gamma_{B}|{\hat{h}}_{RE}{|}^{2})+(2\rho \zeta \bar{\gamma }\left[\right|{\hat{h}}_{RE}{|}^{2}\left(\gamma_{A}+\Phi^{2}\gamma_{J}+1\right)\\ \; & \;+\sigma_{e}^{2}\left(\gamma_{A}+\gamma_{B}+\Phi^{2}\gamma_{J}+1\right)]+\tilde{\rho }\left(\bar{\gamma }+1\right)))\\ \; & \;(2\rho \zeta \bar{\gamma }|h_{AR}{|}^{2}\left(\Phi^{2}\gamma_{J}+1\right)+\tilde{\rho }\left(\bar{\gamma }+1\right)), \end{array} \end{array}$$

(64) $$\begin{array}{c} \begin{array}{cc} \; & z_{IV,B}^{SC}=\\ \; & ((2\rho \zeta \bar{\gamma }\gamma_{A}|{\hat{h}}_{RE}{|}^{2})+(2\rho \zeta \bar{\gamma }\left[\right|{\hat{h}}_{RE}{|}^{2}\left(\gamma_{B}+\Phi^{2}\gamma_{J}+1\right)\\ \; & \;+\sigma_{e}^{2}\left(\gamma_{A}+\gamma_{B}+\Phi^{2}\gamma_{J}+1\right)]+\tilde{\rho }\left(\bar{\gamma }+1\right)))\\ \; & \;(2\rho \zeta \bar{\gamma }|h_{BR}{|}^{2}\left(\Phi^{2}\gamma_{J}+1\right)+\tilde{\rho }\left(\bar{\gamma }+1\right)), \end{array} \end{array}$$

(65) $$\frac{w_{IV}^{SC}}{z_{IV}^{SC}}=\left\{\begin{array}{cc} \frac{w_{IV,A}^{SC}w_{IV,B}^{SC}}{z_{IV,A}^{SC}z_{IV,B}^{SC}}, & C_{S,A}\geq 0\;and\;C_{S,B}\geq 0\\ \frac{w_{IV,A}^{SC}}{z_{IV,A}^{SC}}, & C_{S,A}\geq 0\;and\;C_{S,B}<0\\ \frac{w_{IV,B}^{SC}}{z_{IV,B}^{SC}}, & C_{S,A}<0\;and\;C_{S,B}\geq 0\\ 0, & C_{S,A}<0\;and\;C_{S,B}<0, \end{array}\right.$$

and ${\left(.\right)}_{IV}^{SC}$ denotes the fourth case with SC at the eavesdropper.

## 4. Optimization Problem Formulation

The secrecy capacity optimization problem for MRC and SC at the eavesdropper is

(66) $$\begin{array}{c} \min_{\rho ,\tilde{\rho },P_{A},P_{B},P_{J}}\;\frac{z}{w} \end{array}$$

(67) $$\begin{array}{cc} s.t\;P_{A}+P_{B}+P_{J} & \leq P_{T} \end{array}$$

(68) $$\begin{array}{cc} \rho +\tilde{\rho } & \leq 1 \end{array}$$

(69) $$\begin{array}{cc} \rho ,\tilde{\rho },P_{A},P_{B},P_{J} & \geq 0 \end{array}$$

where *w* and *z* are defined below for each diversity scheme. We exploited the structure of this problem and made use of geometric programming (GP) to jointly optimize the time switching ratio for energy harvesting and the power allocation to the users and jammer to maximize the secrecy capacity.

The standard form of a GP problem is [72]

(70) $$\begin{array}{ccc} \min & f_{0}\left(x\right) & \; \end{array}$$

(71) $$\begin{array}{ccc} s.t & \;f_{i}\left(x\right) & \leq 0,i=1,\ldots ,m, \end{array}$$

(72) $$\begin{array}{ccc} \; & g_{i}\left(x\right) & =0,i=1,\ldots ,p, \end{array}$$

where $f_{i}\left(x\right)$ is a posynomial function, $g_{i}\left(x\right)$ is a monomial function, and *x* is an optimization variable. A monomial function *g* of *x* is a real-valued function of the form $g\left(x\right)=cx_{1}^{a_{1}}x_{2}^{a_{2}}\ldots x_{n}^{a_{n}}$, where c>0, $a_{i}\in R$, and *n* is the number of optimization variables. A posynomial function is the sum of two or more monomials such that $f\left(x\right)=\sum_{k=1}^{K}c_{k}x_{1}^{a_{1k}}x_{2}^{a_{2k}}\ldots x_{n}^{a_{nk}}$, where $c_{k}>0$ and *K* is the number of monomial functions.

The constraints in (67) and (68) are posynomials. This problem can be transformed into GP form and then into a convex problem because the constraints and the objective function are posynomials. However, the objective function is the ratio of two posynomials, so it cannot be transformed into GP form. To solve this problem, $w(\rho ,\tilde{\rho },P_{A},P_{B},P_{J})$ is approximated as a monomial function using the single condensation method (SCM) [72]. In the SCM, the denominator of the ratio of posynomials is approximated with a monomial function. The numerator (a posynomial) is not approximated, hence the term single. In the optimization problem, $w\left(x\right)=\sum_{i}u_{i}\left(x\right)$, where $x={\left[\rho ,\tilde{\rho },P_{A},P_{B},P_{J}\right]}^{T}$ is the sum of *i* monomials, so it is a posynomial by definition. The monomial approximation of w(x) using the SCM is

(73) $$\bar{w}\left(x\right)=\prod_{i}{\left(\frac{u_{i}\left(x\right)}{\alpha_{i}}\right)}^{\alpha_{i}},$$

such that $w\left(x\right)\geq \bar{w}\left(x\right)$. For a given x, $\alpha_{i}$∀i are obtained in w(x) so that

(74) $$\alpha_{i}=\frac{u_{i}\left(x\right)}{w(x)},$$

and $\bar{w}\left(x\right)$ is substituted for w(x) in (66). The objective function after the SCM approximation is a polynomial (posynomial). The key to solving a GP efficiently is to convert it to a nonlinear, but convex optimization problem, which is a problem with a convex objective function, convex inequality constraints, and linear equality constraints. A logarithmic change of variables and a logarithmic transformation of the objective function and constraints are used to obtain a GP form. The resulting problem is convex and can be solved efficiently using CVX [72]. As the optimal solution may be far from the initial guess $x_{0}$ used in the SCM approximation, an iterative approach is used to solve this problem.

For MRC at the eavesdropper, the initial guess is used to calculate $SNR_{E,A}^{\left(1\right)}$, $SNR_{E,A}^{\left(2\right)}$, $SNR_{E,B}^{\left(1\right)}$, and $SNR_{E,B}^{\left(2\right)}$ given by (8), (25), (9), and (27), respectively. $SNR_{E,A}^{\left(1\right)}$, $SNR_{E,A}^{\left(2\right)}$, $SNR_{E,B}^{\left(1\right)}$, and $SNR_{E,B}^{\left(2\right)}$ are then substituted in (29) along with $SNR_{A}$ from (18) and $SNR_{B}$ from (20) to calculate $C_{S,A}$ and $C_{S,B}$, respectively. Then, $C_{S,A}$ and $C_{S,B}$ are compared to determine which case in Section 3.1 to employ, and $x_{0}$ is used to obtain $C_{S,A}$ and $C_{S,B}$. Next, $w_{\left(.\right)}^{MRC}$ is approximated using the SCM, and the resulting ${\bar{w}}_{\left(.\right)}^{MRC}\left(x\right)$ is used in (66) to solve the optimization problem. If the current optimal solution, $x_{k+1}$ satisfies the initial assumption $C_{S,A}\geq 0$ and $C_{S,B}\geq 0$, then $x_{k+1}$ is used to calculate $\bar{w}\left(x_{k+1}\right)$, and the optimization problem is solved again. If $x_{k+1}$ violates $C_{S,A}\geq 0$ and $C_{S,B}\geq 0$, then proceed to the next case. The algorithm to obtain the optimal values ${\left[\rho^{\,*},{\tilde{\rho }}^{\,*},P_{A}^{\,*},P_{B}^{\,*},P_{J}^{\,*}\right]}^{T}$ is summarized in Algorithm 1.

For SC at the eavesdropper, the initial guess is used to calculate $SNR_{E,A}^{\left(1\right)}$, $SNR_{E,A}^{\left(2\right)}$, $SNR_{E,B}^{\left(1\right)}$, and $SNR_{E,B}^{\left(2\right)}$ given by (8), (25), (9), and (27), respectively. The values of $SNR_{E,A}^{\left(1\right)}$ and $SNR_{E,A}^{\left(2\right)}$ are compared to determine which expression for $C_{S,A}$ to consider, and the values of $SNR_{E,B}^{\left(1\right)}$ and $SNR_{E,B}^{\left(2\right)}$ are compared to determine which expression for $C_{S,B}$ to consider. These results determine which case in Section 3.2 to employ. $x_{0}$ is then used to calculate the values of $C_{S,A}$ and $C_{S,B}$. Next, $w_{\left(.\right)}^{SC}$ is approximated using the SCM method, and the resulting ${\bar{w}}_{\left(.\right)}^{SC}\left(x\right)$ is used in (66) to solve the optimization problem. If the current optimal solution, $x_{k+1}$, satisfies the initial assumption $C_{S,A}\geq 0$ and $C_{S,B}\geq 0$, then $x_{k+1}$ is used to calculate $\bar{w}\left(x_{k+1}\right)$, and the optimization problem is solved again. If $x_{k+1}$ violates $C_{S,A}\geq 0$ and $C_{S,B}\geq 0$, then proceed to the next case. The algorithm to obtain the optimal values ${\left[\rho^{\,*},{\tilde{\rho }}^{\,*},P_{A}^{\,*},P_{B}^{\,*},P_{J}^{\,*}\right]}^{T}$ is summarized in Algorithm 2.
**Algorithm 1:** Optimization of the secrecy capacity, $C_{S}$, for MRC at the eavesdropper.**Require:** Channel coefficients, power constraint $P_{T}$, energy conversion efficiency ζ, noise variance $\sigma^{2}$, tolerance ϵ, estimation error variance $\sigma_{e}^{2}$, k=1
1:**while**$|C_{S,k}-C_{S,k-1}|>\epsilon$**do**2: Calculate the monomial approximation $\bar{w}$ for *w* using the single condensation method at $x={\left[\rho_{k},{\tilde{\rho }}_{k},P_{A,k},P_{B,k},P_{J,k}\right]}^{T}$3: k=k+14: Solve the optimization problem in (66) using $\bar{w}$ to find $\left[\rho_{k+1},{\tilde{\rho }}_{k+1},P_{A,k+1},P_{B,k+1},P_{J,k+1}\right]$5: Using the solution in Step 4, calculate $C_{S,A}$ and $C_{S,B}$6: **if** $C_{S,A}\geq 0$ and $C_{S,B}\geq 0$ **then**7:  Go to Step 28: **else**9:  Continue to the next case of $C_{S,A}$ and $C_{S,B}$10: **end if**11: Solve the optimization problem in (32) to obtain $\left[\rho_{k},{\tilde{\rho }}_{k},P_{A,k},P_{B,k},P_{J,k}\right]$12:**end while**13:Assign ${\left[\rho^{\,*},{\tilde{\rho }}^{\,*},P_{A}^{\,*},P_{B}^{\,*},P_{J}^{\,*}\right]}^{T}={\left[\rho_{k},{\tilde{\rho }}_{k},P_{A,k},P_{B,k},P_{J,k}\right]}^{T}$ and $C_{S}=C_{S,k}$


**Algorithm 2:** Optimization of the secrecy capacity, $C_{S}$, for SC at the eavesdropper.**Require:** Channel coefficients, power constraint $P_{T}$, energy conversion efficiency ζ, noise variance $\sigma^{2}$, tolerance ϵ, estimation error variance $\sigma_{e}^{2}$, k=1 **while**
$|C_{S,k}-C_{S,k-1}|>\epsilon$
**do**
2: Calculate $SNR_{E,A}^{\left(1\right)}$, $SNR_{E,A}^{\left(2\right)}$, $SNR_{E,B}^{\left(1\right)}$, and $SNR_{E,B}^{\left(2\right)}$ **if** $SNR_{E,A}^{\left(1\right)}\geq SNR_{E,A}^{\left(2\right)}$ and $SNR_{E,B}^{\left(1\right)}\geq SNR_{E,B}^{\left(2\right)}$ **then**4:  Calculate the monomial approximation $\bar{w}$ for *w* using the single condensation method at $x={\left[\rho_{k},{\tilde{\rho }}_{k},P_{A,k},P_{B,k},P_{J,k}\right]}^{T}$  k=k+16:  Solve the optimization problem in (66) using $\bar{w}$ to find $\left[\rho_{k+1},{\tilde{\rho }}_{k+1},P_{A,k+1},P_{B,k+1},P_{J,k+1}\right]$  Using the solution in Step 7, calculate $C_{S,A}$ and $C_{S,B}$8:  **if** $C_{S,A}\geq 0$ and $C_{S,B}\geq 0$ **then**   Go to Step 110:  **else**   Continue to the next case of $C_{S,A}$ and $C_{S,B}$12:  **end if** **else**14:  Continue to the next case of $SNR_{E,A}^{\left(1\right)}≷SNR_{E,A}^{\left(2\right)}$ and $SNR_{E,B}^{\left(1\right)}≷SNR_{E,B}^{\left(2\right)}$ **end if**16: Solve the optimization problem in (32) to obtain $\left[\rho_{k},{\tilde{\rho }}_{k},P_{A,k},P_{B,k},P_{J,k}\right]$**end while**18:Assign ${\left[\rho^{\,*},{\tilde{\rho }}^{\,*},P_{A}^{\,*},P_{B}^{\,*},P_{J}^{\,*}\right]}^{T}={\left[\rho_{k},{\tilde{\rho }}_{k},P_{A,k},P_{B,k},P_{J,k}\right]}^{T}$ and $C_{S}=C_{S,k}$


## 5. Results and Discussion

In this section, the secrecy capacity is evaluated for a two-way relay network with a friendly jammer in the presence of an eavesdropper. Users *A* and *B* can only communicate through *R* since there is no direct link between them. The simulation parameters were as follows, unless noted otherwise. The noise variance was $\sigma^{2}=10^{-3}$, $\sigma_{e}^{2}=0.1$, T=1; the optimization tolerance was ϵ=0.001, Φ=0; the energy conversion efficiency was ζ=0.5. The channel gains $|h_{AR}{|}^{2}$, $|h_{JR}{|}^{2}$, $|h_{JE}{|}^{2}$, and $|h_{BR}{|}^{2}$ are exponential random variables with mean λ=1, $|h_{RE}{|}^{2}$ and $|h_{BE}{|}^{2}$ are exponential random variables with mean $\lambda_{Eve}$; $|h_{AE}{|}^{2}$ is an exponential random variable with mean $\frac{1}{\lambda_{Eve}}$, $\lambda_{Eve}\in \left\{1,2,3\right\}$. The node locations were normalized to the distance between *A* and *B* so that *A* and *B* were at (0, 0) and (1, 0), respectively. *R* is at the midpoint, (0.5, 0); *J* is at (0.5, −0.5); $P_{T}=10$ dB, and $P_{J}=0.1P_{T}$.

**Figure 3:**
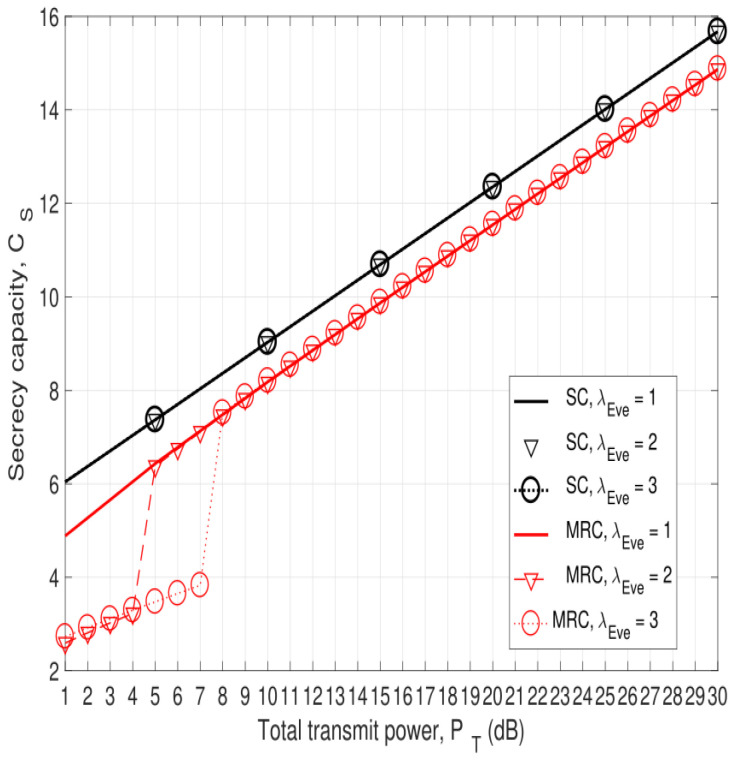
The secrecy capacity versus the total transmit power, $P_{T}$, with $\lambda_{Eve}=1$ and $\sigma_{e}^{2}=0$.

Figure 3 presents the secrecy capacity versus the total transmit power, $P_{T}$, for $\lambda_{Eve}=1$, 2, and 3 with SC and MRC at the eavesdropper. The secrecy capacity increases in all cases as the total transmit power increases. The secrecy capacity of SC outperforms MRC for all values of $\lambda_{Eve}$. The reason is that SC selects only one wiretapped link, which reduces the SNR at the eavesdropper. As a result, the secrecy capacity of the network with SC at the eavesdropper is higher than that with MRC. The effect of increasing $\lambda_{Eve}$ on the secrecy capacity of SC and MRC is negligible except for MRC with $P_{T}\leq 8$ dB. This is because increasing $\lambda_{Eve}$ improves the corresponding link of the eavesdropper, but degrades the other eavesdropper link given the total transmit power $P_{T}$.

**Figure 4:**
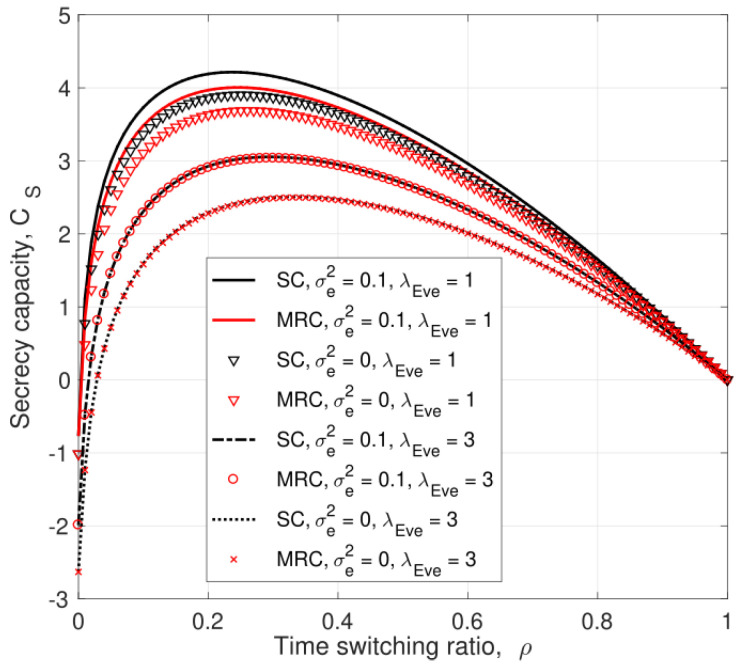
The secrecy capacity versus the time switching ratio, ρ, for different values of $\lambda_{Eve}$ and $\sigma_{e}^{2}$ with $P_{J}=0.1P_{T}$ and $P_{T}=10$ dB.

Figure 4 presents the secrecy capacity versus the time switching ratio, ρ, with SC and MRC for $\sigma_{e}^{2}=0$ and 0.1. This shows that SC outperforms MRC for the given values of $\sigma_{e}^{2}$ and $\lambda_{Eve}$, and the secrecy capacity for imperfect CSI, $\sigma_{e}^{2}=0.1$, is better than that for perfect CSI, $\sigma_{e}^{2}=0$, for all values of ρ. Considering the SNR expressions of the eavesdropper links, the denominators of (8), (25), (9), and (27) contain $\sigma_{e}^{2}$, so increasing this term reduces the SNR at *E*. These results also show that the secrecy capacity increases as ρ increases until it reaches an optimal value, and then, the secrecy capacity decreases. As the time switching ratio increases, the relay harvests more energy for signal forwarding in the second phase. However, a larger ρ means the eavesdropper has more time to overhear the transmitted signals, so there is a tradeoff.

### 5.1. Channel Estimation Error

**Figure 5:**
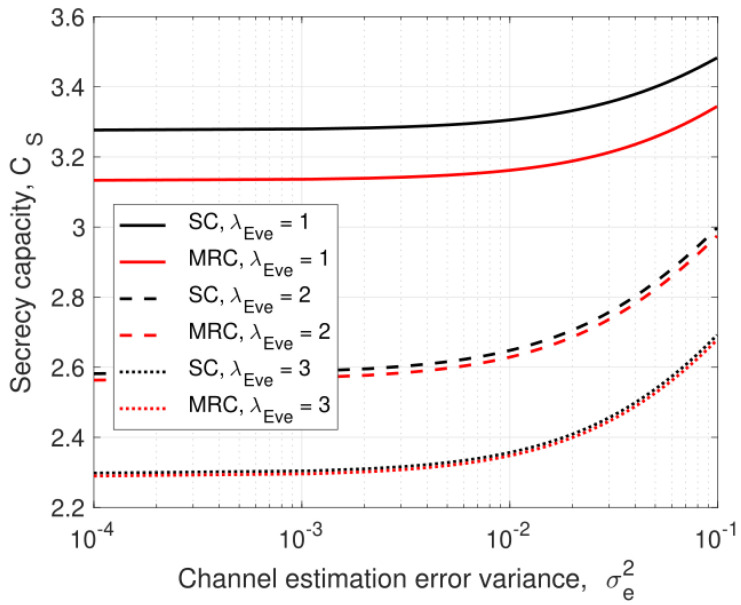
The secrecy capacity versus the channel estimation error variance, $\sigma_{e}^{2}$, for three values of $\lambda_{Eve}$ with ρ=0.5, $P_{J}=0.1P_{T}$, and $P_{T}=10$ dB.

**Figure 6:**
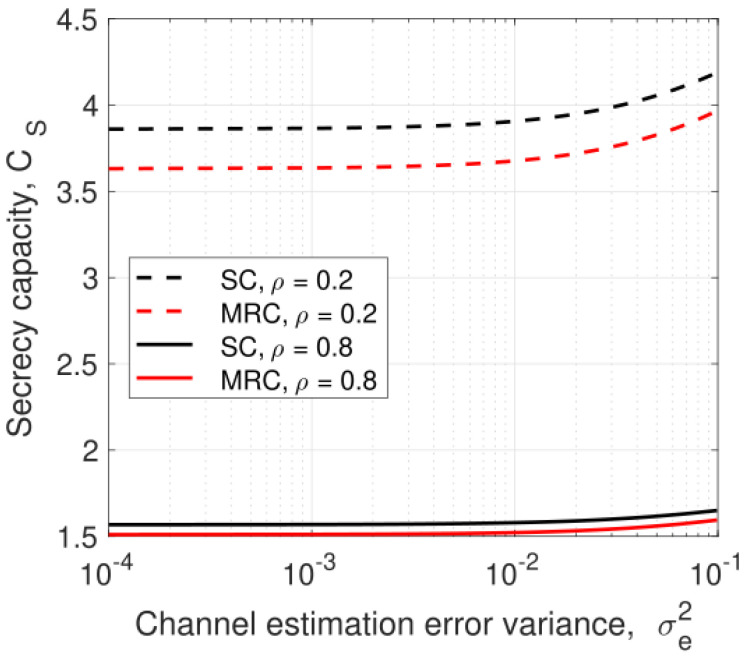
The secrecy capacity versus the channel estimation error variance, $\sigma_{e}^{2}$, with ρ=0.8 and 0.2, $\lambda_{Eve}=1$, $P_{J}=0.1P_{T}$, and $P_{T}=10$ dB.

Figure 5 and Figure 6 present the effect of the channel estimation error variance, $\sigma_{e}^{2}$, on the secrecy capacity. Figure 5 shows the secrecy capacity for $\lambda_{Eve}=1,2,$ and 3. A higher value of $\sigma_{e}^{2}$ means that the eavesdropper is less able to estimate the wiretap links, so the secrecy capacity improves. The differences in secrecy capacity between SC and MRC are 0.14, 0.023, and 0.014 bits/sec/channel use for $\lambda_{Eve}=1,2,$ and 3, respectively, at $\sigma_{e}^{2}=0.1$. Thus, increasing $\lambda_{Eve}$ decreases the gap between SC and MRC. This is because a larger $\lambda_{Eve}$ improves the corresponding link of the eavesdropper, but degrades the other eavesdropper link.

Figure 6 shows the secrecy capacity versus the channel estimation error variance for ρ=0.8 and 0.2 with $\lambda_{Eve}=1$. At $\sigma_{e}^{2}=0.01$, SC outperforms MRC with a difference of 0.057 at ρ=0.8 and 0.23 at ρ=0.2. Thus, decreasing ρ improves the performance of SC and MRC, but does not have a significant effect on the difference between them. As ρ increases, the relay harvests more energy, so there is more transmit power at the relay. This improves the received SNR at the users.

### 5.2. Jammer, Cancellation Factor, and Locations

**Figure 7:**
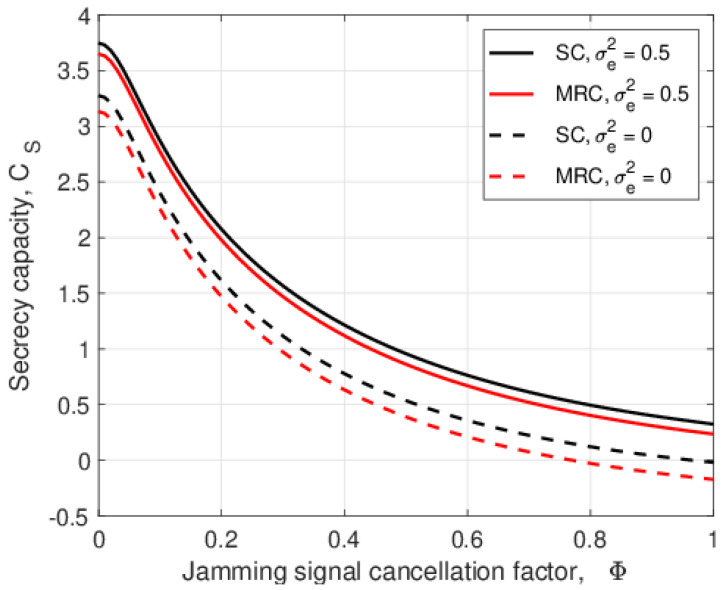
The secrecy capacity versus the jamming signal cancellation factor, Φ, with $\lambda_{Eve}=1$, θ=0.5, $P_{T}=10$ dB, and $P_{J}=0.1P_{T}$.

The secrecy capacity versus the jamming signal cancellation factor, Φ, is given in Figure 7 for $\sigma_{e}^{2}=0$ and 0.5. This shows that SC outperforms MRC for both values of $\sigma_{e}^{2}$. When Φ=0, the secrecy capacity is highest because the jamming signal at the relay is completely cancelled. As Φ increases, more jamming power is amplified and forwarded to *A* and *B*. Thus, the noise at *A* and *B* increases, which degrades their SNRs and, so, decreases the secrecy capacity. The difference in secrecy capacity with SC is 0.93 bits/sec/channel use at Φ=0.1, and this decreases to 0.74 bits/sec/channel use at Φ=0.8.

**Figure 8:**
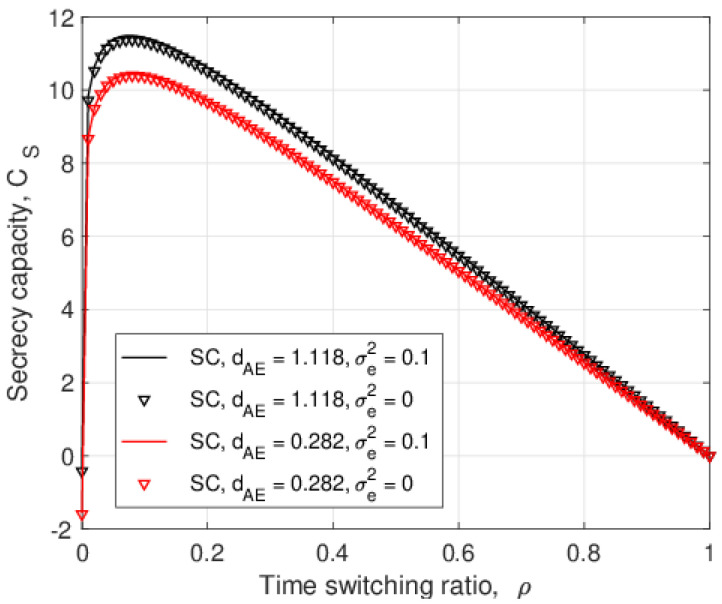
The secrecy capacity for SC at the eavesdropper with $d_{AE}=0.28$ and 1.12, $\lambda_{Eve}=1$, $P_{T}=10$ dB, and $P_{J}=0.1P_{T}$.

**Figure 9:**
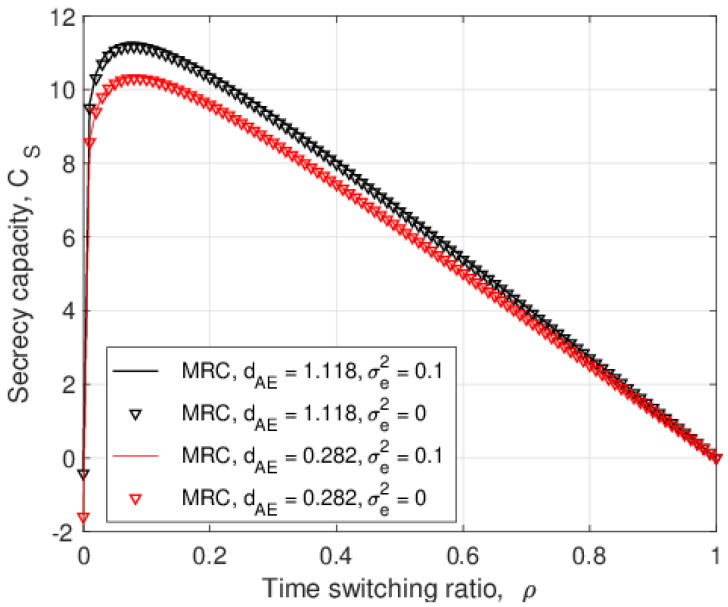
The secrecy capacity for MRC at the eavesdropper with $d_{AE}=0.28$ and 1.12, $\lambda_{Eve}=1$, $P_{T}=10$ dB, and $P_{J}=0.1P_{T}$.

In the following figures, the secrecy capacity is considered for different locations of the eavesdropper and jammer. The channel links can be expressed as $h_{ij}=\frac{f_{ij}}{d_{ij}^{m}}$, where $f_{ij}$ is an exponential random variable with mean =1, m=2.7 is the path loss exponent, and $d_{ij}$ is the distance between *i* and *j*. Figure 8 and Figure 9 present the secrecy capacity versus Φ for SC and MRC at the eavesdropper, respectively. The jammer is at (0.5, −0.5), and the location of the eavesdropper is (0.5, −1) and (0.2, −0.2) with $d_{AE}=1.12$ and 0.28, respectively. These results show that the secrecy capacity increases as $d_{AE}$ increases from 0.28 to 1.12 for both values of $\sigma_{e}^{2}$. The reason is that, as $d_{AE}$ increases, less power is required to be allocated to the jammer. Hence, more power is allocated to *A* and *B*, and more energy is harvested at *R*. Figure 8 shows that, when $\sigma_{e}^{2}=0$, the difference in SC secrecy capacity for $d_{AE}=1.12$ and 0.28 is 0.53 bit/sec/channel use, and this increases to 0.57 bit/sec/channel use for $\sigma_{e}^{2}=0.1$. Figure 9 shows that, when $\sigma_{e}^{2}=0$, the difference in MRC secrecy capacity for $d_{AE}=1.12$ and 0.28 is 0.51 bit/sec/channel use, and this increases to 0.62 bit/sec/channel use for $\sigma_{e}^{2}=0.1$.

**Figure 10:**
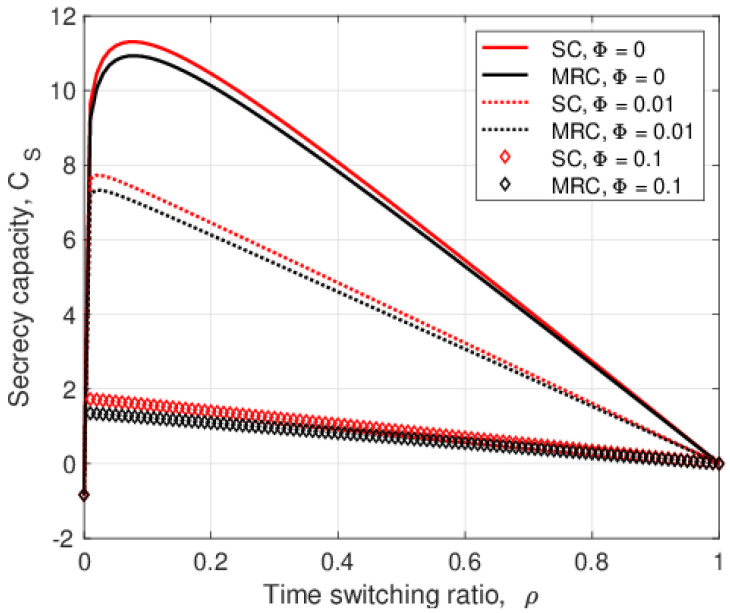
The secrecy capacity for different values of Φ with the jammer at (0.5,−0.1), the eavesdropper at (0.2,−1), $\lambda_{Eve}=1$, $P_{T}=10$ dB, and $P_{J}=0.1P_{T}$.

Figure 10 presents the effect of Φ on the secrecy capacity when the jammer is close to the relay. In this case, *E* is at (0.2,−1) and *J* is at (0.5,−0.1), so significant jamming power is received by the relay. These results show that a small increase in Φ causes a significant drop in secrecy capacity for both SC and MRC. For example, with SC and ρ=0.5, the secrecy capacity for SC drops by 2.74 bit/sec/channel use when Φ increases from 0 to 0.01 and by 3.17 bit/sec/channel use when Φ increases from 0.01 to 0.1. This is because the jamming signal at the relay is larger as the jammer is closer to the relay.

**Figure 11:**
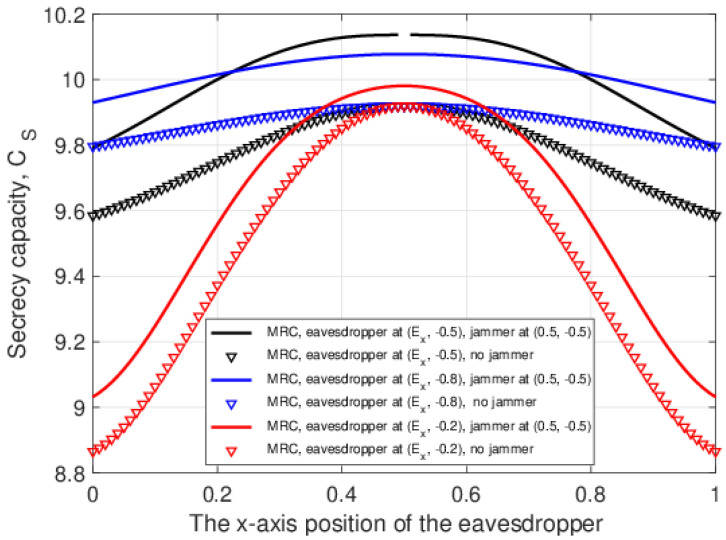
The secrecy capacity versus the *x*-axis location of the eavesdropper (employing MRC), with a jammer at a fixed location and without a jammer.

Figure 11 shows the secrecy capacity versus the *x*-axis location of the eavesdropper (employing MRC), when the jammer is located at (0.5,−0.5) and without a jammer. Results are given for *y*-axis eavesdropper positions −0.2, −0.5, and −0.8 and MRC at the eavesdropper. The solid lines are for the case with a jammer at (0.5,−0.5), and the other lines correspond to no jammer. When the eavesdropper is at x=0.5, i.e., midway between *A* and *B*, the secrecy capacity is the highest. This is because the maximum eavesdropper SNR from *A* and *B* will be smallest at this point. Further, the secrecy capacity is better with a jammer since the jamming signal reduces the SNR at the eavesdropper regardless of their *y*-axis position. The lowest secrecy capacity in both cases (with and without a jammer) is when the eavesdropper is at x=0 and x=1 since the SNR at the eavesdropper from *A* and *B*, respectively, is highest.

**Figure 12:**
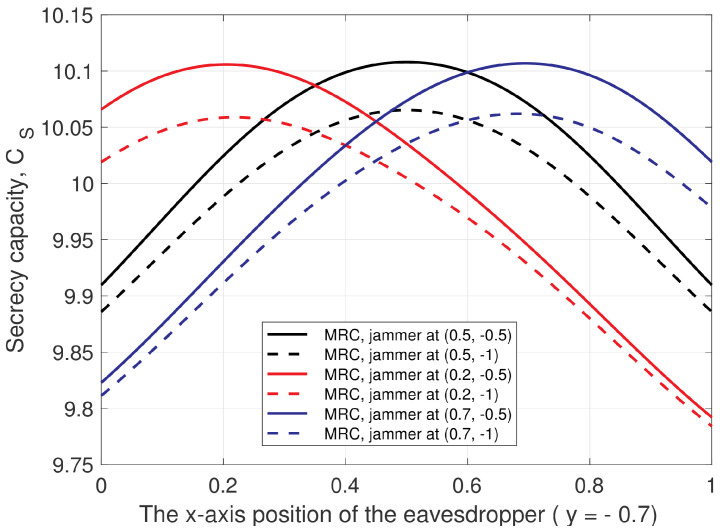
The secrecy capacity versus the *x*-axis location of the eavesdropper (employing MRC), for different jammer locations with $\sigma_{e}^{2}=0$, Φ=0, and $P_{T}=10$ dB.

Figure 12 presents the secrecy capacity versus the *x*-axis position of the eavesdropper (employing MRC), with the jammer located at (0.5,−0.5), (0.5,−1), (0.2,−0.5), (0.2,−1), (0.7,−0.5), and (0.7,−1). The location of the eavesdropper changes from (0,−0.7) to (1,−0.7). In all cases, the secrecy capacity is a minimum when the eavesdropper is at x=0 or x=1, which is closest to *A* or *B*, respectively. As the eavesdropper moves from x=0 to 1, the jamming signal power at the eavesdropper increases and the secrecy capacity increases. Then, the secrecy capacity decreases as the eavesdropper moves farther from the jammer after the maximum secrecy capacity has been reached.

### 5.3. Computational Complexity

Matlab R2017a on a MacBook Pro laptop with an Intel Core i5 processor was used to obtain the simulation results. The average time to run Algorithm 1 (MRC) was 7.89 s and to run Algorithm 2 (SC) was 1.56 s. One reason for this difference is that an iteration of Algorithm 1 requires 648 arithmetic operations, while an iteration of Algorithm 2 requires 318 operations. This is because the number of monomial terms to be approximated with Algorithm 1 is 40, but only 12 with Algorithm 2. Furthermore, the average number of iterations required to solve the optimization problem for Algorithm 1 was 2.63 s and for Algorithm 2 was 1.81 s.

## 6. Conclusions

In this paper, the secrecy capacity was investigated for a two-way energy-constrained time-switching relay network in the presence of an eavesdropper. A friendly jammer was used to reduce the ability of the eavesdropper to intercept the user signals. The secrecy capacity was maximized by jointly optimizing the time switching ratio, ρ, and the transmit power of the two users, *A* and *B*, and the jammer *J*. The single condensation method (SCM) was employed to convert the objective function of the corresponding optimization problem into a posynomial form suitable for geometric programming (GP). Then, GP was used to transform the non-convex objective function to obtain a convex optimization problem. Two diversity combining techniques, MRC and SC, were employed at the eavesdropper. Imperfect cancellation of the jamming signal at the relay was also considered. Results were presented that showed that the imperfect jamming signal cancellation at the relay degrades the secrecy capacity. In addition, utilizing a jammer improves the secrecy capacity and increases the amount of harvested energy at the relay. Further, the secrecy capacity is higher if the jammer is located closer to the eavesdropper. Imperfect channel estimation at the eavesdropper was also investigated. It was shown that, as the estimation error increases, the secrecy capacity improves. MRC has been shown to provide a lower secrecy capacity than SC. Thus, to achieve the SC secrecy capacity with MRC at the eavesdropper, a higher SNR is required at *A* and *B*.

## Data Availability

Not applicable.

## Abbreviations

**Symbol**	**Description**
*A*, *B*	Users
*R*	Relay
*J*	Jammer
*E*	Eavesdropper
$h_{ij}$	Channel between node *i* and node *j*
$|h_{ij}{|}^{2}$	Channel gain between node *i* and node *j*
${\hat{h}}_{iE}$	Estimated channel between *E* and node *i*
$|{\hat{h}}_{iE}{|}^{2}$	Estimated channel gain between *E* and node *i*
$e_{iE}$	Channel estimation error between *E* and node *i*
$\sigma_{e}^{2}$	Channel estimation error variance
$n_{i}$	Additive white Gaussian noise (AWGN) at node *i*
$\sigma^{2}$	AWGN variance
$x_{i}$	Signal transmitted by node *i*
$y_{i}$	Signal received at node *i*
$P_{A}$	Transmit power of node *A*
$P_{B}$	Transmit power of node *B*
$P_{R}$	Transmit power of node *R*
$P_{J}$	Transmit power of node *J*
$P_{T}$	Total power constraint
E[.]	Expected value
$y_{Re}$	Energy harvesting signal at the relay
$y_{Ri}$	Information retrieval signal at the relay
ρ	Time switching (TS) ratio
$E_{H}$	Harvested energy
ζ	Energy conversion efficiency
*T*	Total transmission time
*m*	Path loss exponent
Φ	Jamming signal cancellation factor
$y_{R}$	Information retrieval signal after jamming cancellation
$y_{E}^{\left(1\right)}$	Received signal at *E* in the first phase
$y_{E}^{\left(2\right)}$	Received signal at *E* in the second phase
$SNR_{E,A}^{\left(1\right)}$	SNR at *E* for $x_{B}$ sent to *A* in the first phase
$SNR_{E,B}^{\left(1\right)}$	SNR at *E* for $x_{A}$ sent to *B* in the first phase
$SNR_{E,A}^{\left(2\right)}$	SNR at *E* for $x_{B}$ sent to *A* in the second phase
$SNR_{E,B}^{\left(2\right)}$	SNR at *E* for $x_{A}$ sent to *B* in the second phase
$SNR_{A}$	SNR at *A*
$SNR_{B}$	SNR at *B*
$R_{A}$	Achievable rate at *A*
$R_{B}$	Achievable rate at *B*
$R_{E}^{\left(1\right)}$	Achievable rate at *E* in the first phase
$R_{E}^{\left(2\right)}$	Achievable rate at *E* in the second phase
$R_{E}$	Achievable rate at *E* for both phases
$C_{A}$	Secrecy capacity at *A*
$C_{B}$	Secrecy capacity at *B*
$C_{S}$	Secrecy capacity
